# Nucleolus as a cornerstone linking proliferation and metabolism to cellular responses to stress: involvement of transcription factors MYC and p53

**DOI:** 10.3389/fmolb.2026.1749992

**Published:** 2026-02-16

**Authors:** Аnastasiia Moraleva, Nadezhda Antipova, Pankrat Pavlov, Kira Dobrochaeva, Yury Rubtsov

**Affiliations:** 1 Shemyakin-Ovchinnikov Institute of Bioorganic Chemistry RAS, Moscow, Russia; 2 HSE University, Moscow, Russia

**Keywords:** ARF, mTOR, MYC, nucleolar stress, nucleolus, p53, ribosome biogenesis

## Abstract

The nucleoli are a dynamic membraneless organelles in the nucleus playing a key role in cellular homeostasis. Transcription of rDNA, processing of rRNA, and assembly of the ribosomal subunits occur in nucleoli. Aside from ribosome biogenesis, the nucleolus is also involved in the regulation of other crucial functions, including DNA repair, regulation of cell cycle and apoptosis by mediating nucleolar stress responses. This makes it a key hub participating in regulation of various cellular processes. Given the fact, that protein biosynthesis is directly linked to multiple pathways and depends on ribosome production, it is not surprising that ribosome biogenesis is a centerpiece connecting fundamental cellular processes with each other. Of particular interest is the relationship between the nucleolus, cell cycle, and oncogenesis. In tumor and hyperproliferative cells, an increase in nucleolar size and activity directly correlates with enhanced ribosome biogenesis. This process is mutually controlled by oncogenes of the MYC family and tumor suppressors such as p53 and ARF. MYC plays a central role in regulating DNA transcription, and disrupting of ribosome biogenesis regulation could result in nucleolar stress. It induces activation of p53-dependent and p53-independent checkpoint pathways resulting in cell cycle arrest or apoptosis. In addition to its role in carcinogenesis, impaired ribosome biogenesis is associated with neurodegenerative diseases and ribosomopathies such as Diamond–Blackfan anemia. Thus, understanding the molecular mechanisms of nucleolar functions, and its links with main regulators of the cell cycle and oncogenesis is of great importance. It may help finding novel molecular targets and therapeutic approaches to treat disorders associated with dysregulated ribosome biogenesis and control of proliferation. This review considers the main aspects of nucleolar activity regulation, its role in the cell cycle and diseases, and the therapeutic prospects for targeting these processes.

## 1 Introduction

The nucleolus as a key regulator of cellular metabolism and ribosome biogenesis in normal and transformed cells.

### 1.1 Nucleolus

#### 1.1.1 Common facts and global structure

The nucleolus is formed around chromosomal regions known as Nucleolar Organizer Regions (NORs) containing multiple tandem repeats of rRNA genes. Each gene encodes 47S pre-rRNA, which includes the sequences of 18S, 5.8S, and 28S rRNA as well as transcribed and non-transcribed spacers (ITS, ETS) (Pöll et al., 2009; Wang F. et al., 2013; Henras et al., 2015; Correll et al., 2019). Although the main role of the nucleolus is the synthesis and processing of rRNA, and assembly of ribosomes, it has been shown that only about 30% of its proteins indeed are involved in this process (Andersen et al., 2005; Olson, 2011; King et al., 2024b). RNA-modifying enzymes, chromatin-associated factors, proteins of DNA replication and repair, cell cycle regulators, transcription and splicing factors, kinases and phosphatases, ubiquitin-related proteins, cytoskeletal proteins were also found in the nucleolar proteome (Boisvert et al., 2011; Boisvert et al., 2007) It can be estimated that ∼10% of nucleolar proteins are associated with the cell cycle and differentiation. Of these, about half have not been shown to be involved in ribosome biogenesis. For roughly 30% of nucleolar proteins, the exact function has not been defined yet (https://www.proteinatlas.org/humanproteome/subcellular/nucleoli). This led to realization that the nucleolus is a multifunctional center coordinating major cellular processes or, alternatively, serving as a storage space/container for cellular factors that need to be temporarily excluded from other parts of cell (Pederson, 2011; Iarovaia et al., 2019; Tartakoff et al., 2022; King et al., 2024b). Nucleolar proteins are involved in DNA repair, maintenance of genome integrity, recombination, transcription, and telomere maintenance, as well as in cell cycle regulation, apoptosis, and the export of certain mRNAs and tRNAs (Hein et al., 2013; Quin et al., 2014). In addition, the nucleolus acts as a sensor of environmental conditions, allowing fast reaction of affected/stressed cells to various challenges (Kos-Braun et al., 2017; Yang et al., 2018). Nucleolar activity changes during the cell cycle: it is peaking in the G2 phase, while at the beginning of the prophase, the nucleolus temporarily disassembles to be later re-assembled in daughter cells (Hernandez-Verdun, 2011).

### 1.2 Nucleolar stress

Initially, the term nucleolar stress referred to stressful events that disrupt ribosome biogenesis homeostasis and activate the cellular stress response. However, in recent years, nucleolar stress has been used to describe various impairments in nucleolar morphology and function induced by stressors, which ultimately lead to disruption of cellular homeostasis through activation of p53 or other stress signaling (Yang et al., 2018). Translocation of nucleophosmin (NPM1) has been established as a criterion for nucleolar impairment (Taha and Ahmadian, 2024). A characteristic feature of NPM1 is its dynamic subcellular localization—it undergoes constant shuttling between the nucleolus, nucleoplasm, and cytoplasm, which underlies its multifunctionality (Taha and Ahmadian, 2024). In response to diverse stressful exposures, a rapid and conserved reaction is observed: the translocation of major nucleolar protein NPM1 (Table 1) from the nucleolus to the nucleoplasm. This relocation is an early event in the stress response and precedes the activation of key pathways, such as the accumulation of stabilized p53 (Taha and Ahmadian, 2024). For a long time, it remained unclear what the common molecular mechanism triggering this translocation in response to such diverse stressors might be.

**TABLE 1:** Characterization of nucleolar proteins involved in ribosome biogenesis and stress response.

Protein	Name (Full/alternative)	Role in ribosome biogenesis	Stress sensitivity	Phase separation	Proteostasis
NPM1	Nucleophosmin, B23	The main component of the granular component (GC), participates in the assembly of ribosomes, chaperone for ribosomal proteins, histone chaperone for rDNA.	Translocation from the nucleolus to the nucleoplasm during stress; sensor of oxidative stress (S-glutathionylation)	Participates in LLPS, organizes GC, interacts with IDR-containing proteins and rRNA.	Participates in protein quality control in the nucleolus and sequesters denatured proteins during proteotoxic stress
FBL	Fibrillarin	A key enzyme for rRNA methylation (2′-O-methylation) in DFC, a component of the C/D-box snoRNP.	Regulated by p53; overexpressed/hypomodified in cancer; associated with oncoribosome formation	IDR with RG/RGG repeats is involved in the formation of condensates in DFC.	Not described directly, but may participate in the assembly of RNP complexes
NCL	Nucleolin	Participates in pre-rRNA processing, RNA chaperone in DFC, assembly of the pre-40S subunit	Moves from the nucleolus during stress (DNA damage), participates in DNA repair	Participates in LLPS in the nucleolus in the formation of DFC, interacts with other nucleolar proteins and RNA.	Sequesters denatured proteins in GCs under proteotoxic stress in conjunction with NPM1
Nucleostemin	Nucleostemin	Participates in the early stages of ribosome biogenesis (pre-rRNA processing), regulates stem cell proliferation	Deletion inhibits proliferation and activates p53	Not explicitly described, but probably involved in nucleolar organization	Not described
SURF6	SURF6 (RRP14)	Biogenesis factor pre-60S subunit, involved in processing пре-рРНК	The deletion causes processing defects but does not always activate the p53-dependent response (p53-independent pathway)	Together with nucleophosmin, it participates in phase separation in GC	Not described
EBP2	Epstein–Barr nuclear antigen 1-binding protein 2	Large ribosomal subunit biogenesis factor, stabilizes MYC in the nucleolus, protects against degradation	Mediates the response to MYC-induced ribosome biogenesis and participates in feedback with MYC.	Not described explicitly	Regulates MYC stability and interacts with Fbw7γ ubiquitin ligase
TCOF1	Treacle	A scaffold protein of the fibrillar center (FC), involved in FC architecture and rDNA transcription	Phosphorylated by ATM/ATR upon DNA damage, recruits repair complexes to rDNA, key protein in nucleolar stress	IDR with K/E blocks, participates in LLPS and FC organization	Not described
ARF	p14ARF (human), p19ARF (mouse)	Tumor suppressor, inhibits ribosome biogenesis (suppresses RNA Pol I transcription and rRNA processing), binds MDM2 to activate p53	Activated by oncogenic signals (MYC, Ras), DNA stress; released from the complex with NPM1 under stress	Not described as a member of LLPS, but localized to the nucleolus through interaction with NPM1	Regulated by NPM1 (stability and localization)
PPAN	PPAN (Peter Pan), SSF1	A component of the complex with SURF6 and RRP15, involved in the maturation of the pre-60S subunit	The deletion inhibits apoptosis in a p53-independent manner (BAX stabilization)	Not described explicitly	Not described
RRP15	RRP15	A component of the complex with SURF6 and PPAN, involved in the maturation of the pre-60S subunit	Deletion causes cell cycle arrest in G1/S, increases p21, and in p53-null cells causes a metabolic shift and ROS.	Not described explicitly	Not described

The table summarizes the main functional aspects of proteins associated with nucleolar organization, ribosome biogenesis, stress sensitivity, liquid–liquid phase separation (LLPS) capacity, and proteostasis. Shown are the full/alternative names of the proteins, their role in ribosome biogenesis, response to stress stimuli, involvement in biomolecular condensate formation, and functions in maintaining proteostasis within the nucleolus.

### 1.3 Tumor cells boost nucleolar activity to increase ribosome production

In rapidly dividing cells, including tumor cells, ribosome biogenesis is a major metabolic requirement (Donati et al., 2012). In tumor cells with increased proliferative activity, the signaling pathways controlling cell growth are often hyperactivated, leading to increased rDNA transcription by RNA polymerase I (Tsai and Pederson, 2014). Additionally, changes may occur in the copy number of rDNA or 5S rRNA genes, which also contributes to faster ribosome biogenesis (Wang and Lemos, 2017; Feng et al., 2020; Brown et al., 2022). Due to high demand for ribosomes, tumor cells speed up almost all stages of their formation, including rDNA transcription, rRNA processing, and expression of ribosomal proteins (RPs) (Montanaro et al., 2008; Brown et al., 2022). These processes correlate with activation of oncogenes and suppression of anti-oncogenes (Brown et al., 2022). The canonical proto-oncogene MYC is one of the most powerful drivers of ribosome biogenesis due to its simultaneous ability to stimulate the transcription of rDNA as well as genes encoding ribosomal components and key regulators of ribosome biogenesis (Tsai and Pederson, 2014; Brown et al., 2022). Loss of function of oncosuppressors such as p53 or PTEN can lead to enhanced rDNA transcription, which further stimulates ribosome production (Grewal et al., 2005; Brown et al., 2022). MYC and p53 are considered master regulators which, in contrast to highly specialized transcription factors, can control a broad array of the target genes. Together, they regulate expression of 10%–15% of all transcribed genes and govern multiple cellular responses (Van Riggelen et al., 2010; Ahmadi et al., 2021; Huang et al., 2022).

### 1.4 Layered nucleolar organization is supported by liquid-liquid phase separation

The contemporary model of the nucleolus describes it as a dynamic, hierarchically organized condensate of proteins and RNAs formed through liquid-liquid phase separation (LLPS) (Correll et al., 2019; Dogra and Kriwacki, 2025). Phase separation in nucleolus occurs through interactions of ribosomal and non-ribosomal nucleolar proteins with multiple types of RNA, including pre-rRNA transcripts, small nucleolar RNAs (snoRNAs), and regulatory noncoding RNAs (Dogra and Kriwacki, 2025). Therefore, nucleoli consist of complex multi-component biopolymer fluids with viscoelastic properties, exhibiting features of both solids and liquids (Riback et al., 2023; González-Arzola, 2024). The nucleolus contains distinct layers or internal sub-compartments due to differences in the biopolymer sequence-determined biophysical properties (such as droplet surface tension) of macromolecular components (Feric et al., 2016; González-Arzola, 2024). The mammalian nucleolus is internally organized into at least four concentric sub-compartments: the innermost structure is the fibrillar center (FC), followed by the dense fibrillar component (DFC), then the granular component (GC), while the outermost layer corresponds to a ring of condensed peri-nucleolar chromatin (PC) (Muñoz-Velasco et al., 2025). This LLPS dictates the spatial organization of functionally interconnected sub-compartments, responsible for particular stages of ribosome biogenesis (Dogra and Kriwacki, 2025; Muñoz-Velasco et al., 2025).

The Fibrillar Center (FC) is the innermost and densest phase. The FC contains concentrated clusters of rDNA genes in a transcriptionally inactive state and key transcriptional machinery: RNA polymerase I (Pol I), the transcription initiation Upstream Binding Factor (UBF) and DNA topoisomerase I. Proteins forming FC (e.g., the scaffold protein TCOF1/Treacle) (Table 1) have Intrinsically Disordered Regions (IDRs) rich in lysine (K) and glutamic acid (E), which determine their condensation ability (Jaberi-Lashkari et al., 2023; King et al., 2024a; Dogra and Kriwacki, 2025).

The Dense Fibrillar Component (DFC) surrounds the FC and is the site of active transcription and rRNA processing. Pol I transcription occurs precisely at the FC-DFC boundary (Iarovaia et al., 2019; Stenström et al., 2020). Newly synthesized 47S pre-rRNA is immediately released into the DFC which is enriched with proteins featuring IDRs with RG/RGG repeats (rich in R and G residues), such as fibrillarin (FBL) (Table 1) and small nucleolar ribonucleoproteins (snoRNPs) (Feric et al., 2016; Kim and Kwon, 2021; Dogra and Kriwacki, 2025). DFC condensates are hydrophobic, support high surface tension and are viscoelastic. Site-specific covalent rRNA modifications occur in DFC: 2′-O-methylation (guided by C/D-box snoRNAs) and pseudouridylation (guided by H/ACA-box snoRNAs) (Feric et al., 2016; Kim and Kwon, 2021). Early stages of pre-rRNA processing and its co-transcriptional folding facilitated by RNA chaperones (nucleolin/NCL, DDX21) (King et al., 2024a) and regulatory long non-coding RNAs (lncRNAs) such as SLERT and LoNA also take place in the DFC. Most steps of small ribosomal subunit (pre-40S) assembly and maturation are completed within the DFC (Quinodoz et al., 2025). Recently, a new sub-phase identified at the outer DFC boundary is the peripheral dense fibrillar component (PDFC). It may host the final processing steps related to formation of 3′untranslated regions (3′-UTRs) of rRNAs (Shan et al., 2023; Dogra and Kriwacki, 2025).

The Granular Component (GC) is the largest, most heterogeneous and outermost zone, responsible for final assembly and maturation of ribosomal subunits (Feric et al., 2016; King et al., 2024a; Dogra and Kriwacki, 2025). Its low viscosity, hydrophilicity and Brownian fluid behavior are largely determined by the protein nucleophosmin (NPM1/B23), which mediates LLPS with ribosomal proteins and rRNA. The main stages of large ribosomal subunit (pre-60S) maturation occur in both the DFC and GC, with a directional flow of pre-60S particles towards the GC (Quinodoz et al., 2025), where attachment of ribosomal proteins and assemply quality control are finalized. As maturation concludes, the affinity of ribosomal subunits for NPM1 decreases, allowing passive diffusion to the nucleoplasm for subsequent export to the cytoplasm (Feric et al., 2016; King et al., 2024a).

The Nucleolar Rim (NR) is a border phase at the GC-nucleoplasm interface, serving as an exchange interface for components between the nucleolus and the nuclear space. The NR is thought to tether the nucleolus to chromatin, potentially influencing processes such as cell cycle regulation and ribosome biogenesis (Stenström et al., 2020; Muñoz-Velasco et al., 2025)⁠.

The organization and function of the nucleolus is supported by dynamic LLPS maintained by the continuous rRNA transcription and processing. Properties of IDRs in nucleolar proteins determine their accumulation in particular phases. In addition to K/E-blocks in the FC and RG-repeats in the DFC, D/E-tracts (rich in aspartic and glutamic acids) are hypothesized to create a proton gradient, peaking at the FC/DFC and reaching a minimum in the GC (Feric et al., 2016; King et al., 2024a). This gradient may serve as an additional force for directed transport. Phase separation is driven by differences in surface tension, where more hydrophobic and denser DFC is surrounded by less dense, more hydrophilic GC phase. Nucleolar architecture depends on continuous pre-rRNA synthesis, because inhibition of Pol I transcription leads to phase inversion (Correll et al., 2019).

In addition to organizing nucleolar structures, fluid-phase separation (LLPS) also enables the nucleolus to adapt to cellular changes, including stress. Due to the fluid nature of phase-separated compartments, the nucleolus can rapidly reorganize—assemble or disassemble—depending on ribosome production levels or changes in environmental conditions. For example, under stress caused by DNA damage or transcriptional repression, proteins such as nucleophosmin (NPM1) are able to phase separate in the nucleoplasm, which activates stress response pathways, particularly the p53 signaling (Shin and Brangwynne, 2017; Muñoz-Velasco et al., 2025).

### 1.5 Evolutionary aspects

The fundamental principle underlying nucleolar organization across all evolutionary stages is LLPS. This physicochemical ability of ribonucleoprotein complexes to self-organize into biomolecular condensates is an ancient, deeply conserved property of live (Muñoz-Velasco et al., 2025). In prokaryotes, despite the absence of a defined nucleus, prototypes of nucleolar organization based on the same physical principles already exist. Bacteria form ribonucleoprotein (RNP) granules using LLPS mechanisms (Riback et al., 2020). Moreover, many bacteria exhibit spatial clustering of transcription complexes on ribosomal RNA (rRNA) operons, functionally resembling primitive transcription factories (Jin et al., 2017; Muñoz-Velasco et al., 2025). These data suggest that molecular mechanisms of phase separation-based compartmentalization have ancient roots. Archaea play a special role in the evolution of nucleolus, since comparative genomics identified more profound domain homology between eukaryotic nucleolar factors in the archaeal protein domains, than in bacteria (Staub et al., 2004; Riback et al., 2020; Muñoz-Velasco et al., 2025). In some archaea, such as *Sulfolobus solfataricus*, electron-dense regions enriched in RNA and proteins are found within the nucleoid. These structures morphologically resemble the fibrillar component of the eukaryotic nucleolus and can be considered proto-nucleoli, reflecting the initial stages of ribosome biogenesis compartmentalization (Islas-Morales et al., 2023; Muñoz-Velasco et al., 2025).

Emergence of the eukaryotic cell and segregation of transcription from translation necessitated the efficient assembly and export pipeline for ribosomes. Simultaneously, the massive increase in rDNA gene copy numbers and the complexity of their regulatory regions (intergenic spacers, IGS) required developing highly organized space for coordinated regulation of rDNA activity. An important evolutionary step was the emergence and proliferation of specific proteins with long intrinsically disordered regions (IDRs), capable of multivalent, weak interactions, enabling existence of stable, dynamic, and functionally specialized multiphase condensates (González-Arzola, 2024).

The early eukaryotic nucleolus was likely two-layered, consisting of a central fibrillar zone and a peripheral granular component (GC) (González-Arzola, 2024). Further evolution, especially in amniotes (reptiles, birds, mammals), was accompanied by significant complexity (Thiry and Lafontaine, 2005; Lamaye et al., 2011). The elongation of non-coding spacers (ITS/ETS) in rDNA genes and the emergence of new stages of rRNA modification and processing led to the functional and morphological division of the fibrillar zone. It split into two separate compartments: the fibrillar center (FC) and the dense fibrillar component (DFC). The formation of the FC became possible with the appearance of specialized scaffold proteins with IDRs, such as TCOF1 (Treacle) (Jaberi-Lashkari et al., 2023; Lafontaine, 2023; González-Arzola, 2024). In parallel, proteins like nucleophosmin (NPM1) evolved to organize and regulate the GC. This division optimized the conveyor-belt processing of pre-rRNA.

The most significant transformation was the evolution of the nucleolus from a specialized ribosome factory into a multifunctional sensory and regulatory hub. This occurred through the massive incorporation of hundreds of additional proteins into its proteome that are not directly involved in ribosome biogenesis but are linked to cell cycle control, stress response, DNA repair, and apoptosis. The evolution of stress-sensitive pathways, such as the RPL11/MDM2/p53 pathway, created a direct molecular link between nucleolar integrity and the fate of the entire cell, turning the nucleolus into a powerful regulator of cellular homeostasis and tumor suppression.

### 1.6 ncRNAs

Contemporary understanding reveals the nucleolus as a hub for the production and regulation of a wide spectrum of non-coding RNAs (ncRNAs), which form a complex regulatory network, perform diverse functions (Stochaj and Weber, 2020; Han and Chen, 2024; Dogra and Kriwacki, 2025). For example, pRNA and PAPAS (the latter can even be translated into a protein) participate in the repression of rDNA transcription in response to stress (Böğürcü-Seidel et al., 2023; Zhang et al., 2023; Han and Chen, 2024; Wadsworth et al., 2024). LETN facilitates NPM1 pentamerization, critical for GC stability (Böğürcü-Seidel et al., 2023) SLERT, in ultralow stoichiometry, allosterically regulates the helicase DDX21, stimulating Pol I transcription (Xing et al., 2017). LoNA and circANRIL modulate rRNA processing, acting as platforms or inhibitors for complex assembly (McCool et al., 2020). MicroRNAs (miRNAs) perform fine-tuned regulation, integrating oncogenic signals. For instance, miR-504 targets p53, creating a feedback loop that attenuates ribosome biogenesis under oncogenic stress. miR-24, -145, −130a mediate repression of the oncogene MYC, and for this, they require assistance from ribosomal proteins (RPL11, RPL5, RPS14), directly linking nucleolar stress to the control of pro-oncogenic pathways (Challagundla et al., 2011; Liao et al., 2013; Li et al., 2015; McCool et al., 2020). The nucleolus also serves as a site for partial assembly or processing of other ribonucleoprotein complexes, such as telomerase, U6 small nuclear RNA, the signal recognition particle (SRP), and RNase MRP, underscoring its role as a hub for fundamental cellular processes (Feng and Manley, 2022; Böğürcü-Seidel et al., 2023). Thus, the nucleolus represents a unique and highly complex system where the fundamental physical principle of phase separation, evolutionarily inherited from prokaryotes, was co-opted and hypertrophied by eukaryotes to create a highly organized production and regulatory pipeline. Its five-phase dynamic architecture directly reflects and determines the stages of ribosome biogenesis. However, its function extends far beyond this scope. The nucleolus through nucleolar stress signaling and extensive protein and RNA networks integrates and coordinates, diverse processes such as cell cycle control, stress response, genome stability maintenance, apoptosis regulation, and oncogenesis. It acts as a central processor for cellular homeostasis, converting diverse internal and external signals into coordinated structural and functional adaptations.

The table summarizes the main functional aspects of proteins associated with nucleolar organization, ribosome biogenesis, stress sensitivity, liquid–liquid phase separation (LLPS) capacity, and proteostasis. Shown are the full/alternative names of the proteins, their role in ribosome biogenesis, response to stress stimuli, involvement in biomolecular condensate formation, and functions in maintaining proteostasis within the nucleolus.

### 1.7 Aberrant nucleolar organization in pathology

Under normal conditions, the nucleolus exists in a dense, dynamic, liquid-like state that facilitates efficient ribosome biogenesis. However, various cellular stresses can induce nucleolar solidification, leading to a transition towards a more solid-like state. Dysregulation of nucleolar phase transitions is implicated in the pathogenesis of several diseases, including neurodegenerative disorders and cancer (Corman et al., 2023; Dogra and Kriwacki, 2025). Such alterations have been observed in neurodegenerative diseases, including Alzheimer and Huntington, amyotrophic lateral sclerosis (ALS), and frontotemporal dementia (FTD), suggesting a common mechanism of neuronal dysfunction and death (Corman et al., 2023; Dogra and Kriwacki, 2025). A fundamental aspect of nucleolar dysfunction in neurodegeneration is disruption of phase separation and the resulting effects on the nucleolus’s content and properties (Yang et al., 2018; Dogra and Kriwacki, 2025). Stress, triggered by the inhibition of ribosome biogenesis as well as by DNA damage, hypoxia, oxidative stress, heat and cold shock, and nutrient/metabolic stress, often leads to exit of nucleolar proteins to the nucleoplasm (Yang et al., 2018; Dogra and Kriwacki, 2025). Cancer cells often have more numerous nucleoli compared to normal cells (Corman et al., 2023; Dogra and Kriwacki, 2025). These changes reflect underlying alterations in nucleolar organization and dynamics, including aberrant distribution of nucleolar proteins, changes in the spatial arrangement of nucleolar compartments, and modified phase separation properties of the nucleolus.

Concluding this section, it should be noted that the nucleolus is a dynamic and evolutionarily conservative structure that plays a key role not only in ribosome biosynthesis, but also in regulation of cell growth, proliferation, and response to external signals. Further, we will discuss the impact of the most profound regulators of cell proliferation and transcription on the ribosome biogenesis and other nucleolar functions focusing on those which are responsible for reactions to various stresses.

## 2 Regulation of nucleolar activity by the MYC family proteins

### 2.1 Common facts

MYC family oncoproteins (C-myc, N-myc, and L-myc) are involved in the regulation of ribosome biogenesis, mRNA translation, cell cycle, stress responses. They participate also in proliferation, differentiation, and apoptosis in eukaryotic cells (Pelengaris et al., 2002; Ahmadi et al., 2021). MYC is involved in the transition of cells from G1 to S phase, playing a critical role in cell cycle progression. Elevated MYC expression promotes entry to S-phase and stimulates mitosis, even in the absence of growth factors (Ahmadi et al., 2021). High level of MYC boosts the proportion of cells in S and G2/M phases in a dose-dependent manner, accelerates proliferation and promotes increase of cell size. MYC is repressed in a p53-dependent manner in response to DNA damage. In MYC-deficient cells, kinase activity of CDK4, CDK6, and CDK2 is decreased, while G1 and G2 phases are prolonged, emphasizing MYC’s impact on cell cycle regulation (Ahmadi et al., 2021). One of MYC’s key functions is coordinating the production of molecular components required for ribosome assembly. This is especially important for increasing protein synthesis in rapidly growing cells (Derenzini et al., 2017; Penzo et al., 2019). Studies have shown that MYC-dependent cancers often exhibit hyperactivation of ribosome biogenesis, highlighting the role of MYC (Barna et al., 2008). Several mechanisms modulate MYC-dependent ribosome biogenesis regulation under different growth conditions. Signaling pathways, including WNT, SRC, ERK, and Notch, increase MYC expression, while TGF-β signaling tends to reduce MYC activity in mammalian cells (Lourenco et al., 2021; Ni and Buszczak, 2023).

**FIGURE 1:**
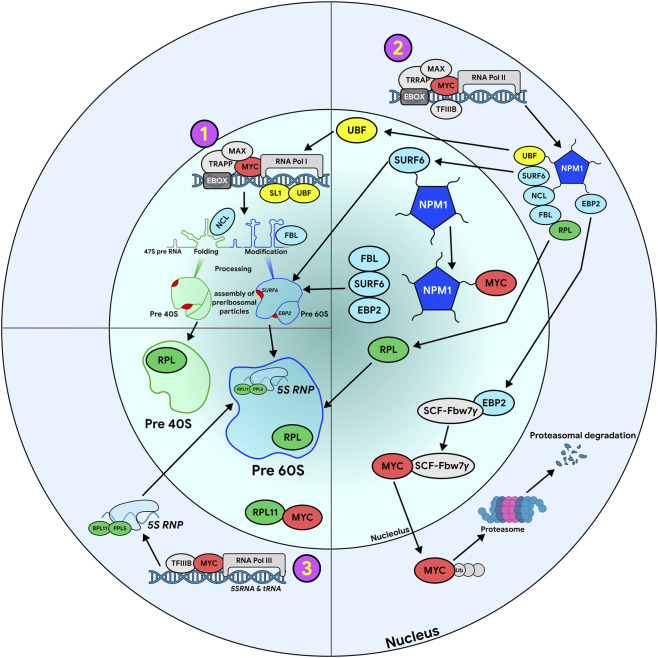
MYC enhances ribosome biogenesis, by regulating transcriptional activity of RNA Pol I, II, and III. Schematic drawing represents events that occur in the nucleus (outer circle) and nucleolus (inner circle). MYC influence on activity of different polymerases is indicated by pink circles with corresponding yellow numbers inside. Below we provide a brief description of events and functions displayed in the figure: 1. Heterodimer of MYC-MAX promotes rDNA transcription by interacting with SL1 augmenting recruitment of Pol I to rDNA promoter region. In addition, MYC binds transcriptional cofactor TRRAP promoting histone acetylation, opening of chromatin and activating rDNA transcription. The synthesized rRNA co-transcriptionally binds to ribosome biogenesis factors and undergoes folding, processing, and modifications; 2. MYC and nucleolar proteins mutually regulate each other. MYC stimulates the transcription of genes involved in ribosomal biogenesis (fibrillarin - FBL, nucleophosmin -NPM1, Surfeit locus protein 6 - SURF6, ribosomal proteins - RPL) (Table 1) with Pol II. The main E3 ubiquitin ligase responsible for MYC degradation in the nucleolus is SCF-Fbw7γ which requires EBP2 protein for nuclear localization, binding with MYC and its polyubiquitination followed by proteosomal degradation. NPM1 maintains nucleolar organization, brings together and anchors nucleolar proteins facilitating their trafficking interactions and functioning. For instance, NPM1 is involved in MYC-mediated stimulation of rRNA synthesis; 3. MYC activates 5S rRNA and tRNA transcription via Pol III by directly engaging TFIIIB. Stimulation of Pol III transcription by MYC can be suppressed by protein RPL11 (Oskarsson and Trumpp, 2005; Van Riggelen et al., 2010).

MYC proteins possess high degree of structural homology, including several key elements: basic region (BR), helix-loop-helix (HLH) motif, and leucine zipper (LZ) at the C-terminus, as well as three highly conserved regions - MYC boxes 1–3 (MB 1–3) - located at the N-terminus (Meyer and Penn, 2008; Ahmadi et al., 2021). BR, HLH, and LZ motifs are required for formation of the MYC/Max heterodimer (Meyer and Penn, 2008; Ahmadi et al., 2021). The MYC/Max complex binds to the E-box motifs (CACGTG) in regulatory/promoter regions of target genes activating their transcription (Meyer and Penn, 2008; Ahmadi et al., 2021). Accumulation of MYC at gene promoter regions increases their transcription (Nie et al., 2012; Ahmadi et al., 2021) (Figure 1). Besides Max, other coactivators are also important for MYC’s interaction with chromatin (Thomas et al., 2015; Lorenzin et al., 2016; Richart et al., 2016; Bumpous et al., 2023). For example, WD repeat-containing protein 5 (WDR5), working as MYC cofactor, is a key regulator of genes encoding proteins responsible for protein biosynthesis. It directly binds to a conserved region within MYC known as Myc Box IIIb and facilitates the recruitment of c-MYC to specific target genes involved in translational processes (Thomas et al., 2015; 2019; Weissmiller et al., 2019; Borgenvik et al., 2021; Bumpous et al., 2023; Liu et al., 2023). According to existing model, MYC possibly does not activate transcription *de novo*, but rather amplifies the expression of transcriptionally active genes, boosting the output of the pre-existing transcriptional program (Lin et al., 2012; Nie et al., 2012; Campbell and White, 2014). Mechanistically, MYC interacts with regulatory factors thereby modulating RNA polymerase activity, globally affecting the rate of transcription.

One of the central functions of MYC, which is critically important for maintaining rapid cell growth, is the global coordination of the production of all ribosomal components. To achieve this, MYC selectively enhances transcription by all three nuclear RNA polymerases, with the greatest impact on rRNA synthesis (Figure 1).

### 2.2 Regulation of transcription by RNA polymerase I (Pol I)

RNA polymerase I (Pol I) is an enzyme responsible for 47S pre-rRNA transcription which needs several cofactors to start transcription. Cofactor UBF binds two rDNA promoter regions: the upstream control element (UCE) and the core promoter. UBF facilitates pre-initiation complex assembly and chromatin remodeling, and promotes the transition from transcription initiation to elongation (Ahmadi et al., 2021). Another companion of Pol I is selective factor 1 (SL1), composed of the TATA-box-binding protein (TBP) and three Pol I-specific TBP-associated factors (TAF1A, TAF1B, TAF1C) which stabilizes UBF binding and formation of pre-initiation complex at the rDNA promoter (Van Riggelen et al., 2010). The third essential factor is Rrn3 (or TIF-1A) interacting with the UBF/SL1 complex and recruiting Pol I to initiate transcription (Campbell and White, 2014). MYC interacts with SL1 components TBP and TAFs, promoting Pol I binding to rDNA promoters (Figure 1) (Van Riggelen et al., 2010), and enhances expression and recruitment of Pol I cofactors UBF, SL1 and Rrn3 (Grandori et al., 2005; Russell and Zomerdijk, 2006; Van Riggelen et al., 2010).

MYC promotes ribosomal DNA (rDNA) transcription by various means (Figure 1). Inside nucleoli, MYC/Max dimers bind to rDNA along with RNA polymerase I (Pol I) (Oskarsson and Trumpp, 2005). This interaction recruitsthe histone acetyltransferase Transformation/Transcription-Associated Protein (TRRAP) increasing acetylation of histones H3 and H4 at the rDNA promoter. It helps opening chromatin, Pol I binding and activating rDNA transcription (Poortinga et al., 2011; Campbell and White, 2014). Furthermore, MYC influences higher-order chromatin structures by promoting the formation of rDNA loops that enhance transcriptional re-initiation by bringing together promoter and terminator (Shiue et al., 2009; Campbell and White, 2014). MYC-mediated activation of Pol I transcription is independent of cell cycle stage, as it could be observed in quiescent cells (Poortinga et al., 2004; Campbell and White, 2014). Beyond UBF, MYC may also influence the expression of other Pol I-specific subunits and factors (Pelengaris et al., 2002; Campbell and White, 2014). Thus, MYC plays critical role in regulating ribosome biogenesis affecting both chromatin and transcription factors. While activation of rDNA genes is considered an ancient function of MYC, direct regulation of Pol I transcription likely evolved later. For example, human and rat rDNA contain several E-boxes, but their number and location are not conserved (Shiue et al., 2009; Campbell and White, 2014). In *Drosophila*, rDNA lacks canonical E-boxes, and dMyc binding to rDNA has not been detected (Grewal et al., 2005; Campbell and White, 2014).

### 2.3 Regulation of transcription by RNA polymerase III (Pol III)

5S rRNA is synthesized outside the nucleolus by Pol III. MYC affects Pol III gene transcription by interacting with components of transcription factors TFIIIB or TFIIIC depending on cell type (Koch et al., 2007; Campbell and White, 2014). It has been suggested, that TFIIIC can bind MYC associated with histone acetyltransferases facilitating TFIIIB binding to promoter. Moreover, MYC may enhance the expression of Pol III subunits and cofactors, for example, it stimulates transcription of TFIIIB gene, the factor which is necessary for Pol III activity (Figure 1) (Oskarsson and Trumpp, 2005; Van Riggelen et al., 2010). MYC regulates of Pol I and Pol III in similar manner, which is logical since their production has to be coordinated to support MYC-driven cell growth.

### 2.4 RNA polymerase II (Pol II) transcription

Through the canonical E-box binding, MYC activates genes encoding ribosomal proteins (RPs), as well as multiple accessory factors of ribosome biogenesis and function, such as nucleophosmin (NPM1/B23), EIF4E, nucleolin, fibrillarin, SURF6 (Table 1), and many others (Costanzo et al., 2010; Perna et al., 2012; Kodiha and Stochaj, 2013; Li and Hann, 2013; Lafita-Navarro et al., 2018; Hao et al., 2019; Destefanis et al., 2020; López et al., 2020; Brown et al., 2022; Issa et al., 2024). Thus, MYC acts as a central coordinator, simultaneously enhancing production of for ribosomal RNAs, proteins and biogenesis factors. It ensures the balanced production of components needed for efficient ribosome assembly, accelerated protein synthesis and MYC-driven proliferation. The fact that MYC increases in mouse promyelocytes expression of ∼80% of genes involved in Pol I transcription, including UBF and Rrn3, about half of Pol III subunits and co-factors, and only 14% for Pol II (Poortinga et al., 2011; Campbell and White, 2014), supports central role of MYC in regulation of ribosome biogenesis and translation output.

### 2.5 Control of MYC level and activity

MYC amount is controlled at several levels: transcriptional, mRNA life time, translational and post-translational (protein stability). C-myc has short half-life, between 20 and 30 min, due to rapid degradation by ubiquitin-proteasome system (Thomas and Tansey, 2011; Farrell and Sears, 2014; Ahmadi et al., 2021). In tumors, MYC regulation is often disrupted, leading to its abnormally high transcriptional activity (Ruggero, 2009; Dang, 2012; Liao P. et al., 2014; Stine et al., 2015; Dejure and Eilers, 2017; Destefanis et al., 2020; Dong et al., 2020; Ahmadi et al., 2021; Das et al., 2023; Zacarías-Fluck et al., 2024).

Nucleolar components affect MYC levels and activity as well, closing a positive feedback loop linking MYC activity and ribosome assembly (Li and Hann, 2013). Ribosomal Protein 11 of Large subunit (RPL11) binds to MYC and suppresses its transcriptional activity (Li and Hann, 2013), while RPL5 promotes degradation of MYC mRNA by interacting with its 3′UTR via the RNA-induced silencing complex (RISC complex) (Liao J. M. et al., 2014; Stępiński, 2018). The interaction of RPL11 with Mouse Double Minute 2 homolog (MDM2, E3 ubiquitin-protein ligase) acts as a checkpoint for excessive production of ribosomal proteins caused by c-MYC overexpression (Macias et al., 2010).

NPM1 - a major component of the granular fraction of the nucleolus (Kodiha and Stochaj, 2013; Li and Hann, 2013; López et al., 2020; Issa et al., 2024) - represents a particularly critical MYC target, as its function not only provides a structural scaffold for ribosome assembly but also establishes a vital positive feedback loop that directly enhances MYC’s transcription and function and, ensuring coordinated ribosome production. NPM1 acts as a chaperone, preventing protein aggregation in a dense environment of the nucleolus (Figure 1) (Ziv et al., 2014), and helps maintaining nucleolar structure by anchoring other nucleolar proteins (Gibbs et al., 2020; Falini et al., 2025). Its pentameric structure with flexible arms enables NPM1 interaction with numerous molecules, including rRNA, the tumor suppressor p14^ARF^- (ARF,-Alternate open Reading Frame) (Table 1), ribosome biogenesis factors such as fibrillarin, nucleolin, and SURF6, proteins IDRs (Ferrolino et al., 2018; Gibbs et al., 2020). As a part of these aggregates, NPM1 participates in liquid–liquid phase separation (LLPS), formation of nucleolar substructures, and directional transport of maturing pre-rRNAs to the periphery of the nucleolus (Falini et al., 2025). NPM1 regulates rDNA transcription acting as a histone chaperone (Frottin et al., 2019; Falini et al., 2025) and is involved in cellular response to DNA damage (Ogawa and Baserga, 2017; López et al., 2020; Sekhar and Freeman, 2023; Falini et al., 2025). It is believed, that this protein is required for MYC-mediated stimulation of rRNA synthesis (Li and Hann, 2013). NPM1 knockdown in leukemia cells arrests cell cycle in G1 phase through upregulation of p21 and inhibition of the CDK2/Cyclin E complex (Luchinat et al., 2018; Lin et al., 2019). In mouse embryonic fibroblasts, NPM1 forms binary complexes with c-MYC and binds to the promoters of MYC target genes encoding mRNAs and rRNA (Li et al., 2008; Li and Hann, 2013; Luchinat et al., 2018).

Epstein–Barr nuclear antigen 1-binding protein 2 (EBP2) (Table 1), biogenesis factor for the large ribosomal subunit, i s a major player in MYC post-translational regulation. EBP2 binds to MYC promoting its localization to the nucleolus and protecting it from ubiquitination and degradation (Figure 1). This, in turn, enhances rRNA expression and stimulates cell proliferation (Liao P. et al., 2014) (Figure 1). The main E3 ubiquitin ligase responsible for MYC degradation in the nucleolus is F-box and WD repeat domain-containing protein 7, gamma isoform (SCF-Fbw7γ) (Figure 1) (Bonetti et al., 2008; Zhou et al., 2015). EBP2 interacts also with Fbw7γ, ensuring its nucleolar localization (Welcker et al., 2011; Li and Hann, 2013; Liao P. et al., 2014). EBP2 is a direct MYC transcriptional target, forming a positive feedback loop that promotes tumor cell proliferation. EBP2 may also stabilize MYC through an Fbw7-independent mechanism, which deserves further study (Liao P. et al., 2014).

MYC may also promote apoptosis indirectly by increasing p53 levels, which, in turn, suppresses MYC expression. In cancer, this interplay between p53 and MYC is often disrupted (Ahmadi et al., 2021) allowing MYC dual role in tumor cells. Depending on conditions, it can activate or suppress main signaling pathways controlling balance between proliferation and apoptosis (Nie et al., 2012; Ahmadi et al., 2021).

Thus, MYC and nucleolar components mutually regulate each other, ensuring coordinated control of ribosome biogenesis and cell proliferation. However, when these regulatory mechanisms are disrupted, MYC may drive excessive rRNA production, contributing to elevated ribosome biogenesis in tumor cells. This highlights the importance of tightly regulated MYC activity for maintaining normal cell growth and preventing malignant transformation.

## 3 mTOR kinase in response to stresses, regulation of ribosome biogenesis, protein translation and cell proliferation

One of the central regulators of cell growth and adaptation to various stresses is mTOR kinase (mammalian target of rapamycin) (Liu and Sabatini, 2020; Ni and Buszczak, 2023). mTOR is a serine/threonine kinase from PI3K-related kinase family (Liu and Sabatini, 2020; Ni and Buszczak, 2023) which integrates both internal and external signals and coordinating diverse cellular processes, including cell growth, proliferation, and survival (Liu and Sabatini, 2020; Saba et al., 2021). It controls ribosome biogenesis and protein synthesis depending on external conditions and metabolic state of the cell. In addition, mTOR is involved in cellular responses to amino acid deprivation and oxidative stress, thus ensuring cell survival and function under changing conditions (Marini et al., 2018). mTOR forms two multiprotein complexes—mTORC1 and mTORC2 (Liu and Sabatini, 2020; Ni and Buszczak, 2023). mTORC1 mainly regulates cellular anabolism in response to nutrient and growth factor availability (Deleyto-Seldas and Efeyan, 2021). Downstream effectors of mTOR are the kinases S6K1 and S6K2 (Lai et al., 2010), which phosphorylate key substrates such as rpS6, IRS-1, and eIF4B, which promotes global translation and cell growth (Lai et al., 2010). mTORC1 also regulates the translation and function of multiple transcription factors such as hypoxia-inducible factor 1α (HIF1α) and c-MYC (Pourdehnad et al., 2013; Dodd et al., 2015; He et al., 2025).

mTORC1 signaling network affects various stages of ribosome biogenesis, including rRNA transcription, ribosomal protein synthesis, and assembly of functional ribosomes (Chauvin et al., 2014; He et al., 2025). mTORC1 promotes the translation of mRNAs containing a terminal oligopyrimidine (TOP) motif. The TOP motif is found in transcripts of all 79 human ribosomal protein genes, whose translation is tightly regulated and largely dependent on mTORC1 activity (Thoreen et al., 2012; Tao et al., 2020). mTOR and its effector kinase p70-S6 (S6K) control rRNA transcription by phosphorylating the initiation factor TIF-IA, a core component of the Pol I. Phosphorylation of TIFIA modulates both the nucleolar localization and activity of TIF-IA enhancing its interaction with Pol I and rate of rRNA synthesis (Mayer et al., 2004; Ni and Buszczak, 2023). Under nutrient-rich conditions, TIF-IA actively binds to Pol I and localizes to the nucleolus. However, inhibition of mTORC1 disrupts this interaction, accompanied by changes in TIF-IA phosphorylation (Mayer et al., 2004; Ni and Buszczak, 2023). In parallel, the mTORC1–S6K axis by phosphorylation regulates the activity UBF (Ni and Buszczak, 2023). mTOR-dependent activation increases UBF binding to SL1, another component of the Pol I pre-initiation complex, further enhancing rRNA transcription (Stefanovsky et al., 2006; Tao et al., 2020).

In addition to rRNA transcription, mTORC1 controls 5S rRNA synthesis by Pol III. First, mTORC1 phosphorylates and inactivates MAF1, main repressor of Pol III (Kantidakis et al., 2010; Michels et al., 2010; Shor et al., 2010; He et al., 2025). Second, mTORC1 directly promotes assembly of the Pol III complex by facilitating interactions between TFIIIB and TFIIIC (Mayer and Grummt, 2006). It was shown that mTORC1 may directly associate with TFIIIC, further enhancing 5S rRNA transcription (Kantidakis et al., 2010; Michels et al., 2010; Ni and Buszczak, 2023). Independent of effects on Pol I transcription, mTOR can modulate pre-rRNA processing (Iadevaia et al., 2014; Ni and Buszczak, 2023). By activating S6K1 and the ribosomal protein RPS6, mTORC1 enhances production of 40S ribosomal subunits thereby promoting cell growth (Jiao et al., 2023).

Notably, the mTORC2 complex component RICTOR and the enzyme L-glutamine synthetase (GLUL) have recently been identified in screens for human ribosome biogenesis factors (Badertscher et al., 2015; Bohnsack and Bohnsack, 2019). Since the impact of GLUL on 40S subunit biogenesis appears to be independent of mTORC1, intracellular glutamine synthesis may be essential for efficient ribosome production (Badertscher et al., 2015; Bohnsack and Bohnsack, 2019). mTOR also controls translation, enabling regulation of protein synthesis under stress conditions such as amino acid deficiency and oxidative stress. mTOR influences both global and selective mRNA translation (Thoreen et al., 2012; Marini et al., 2018). Taken together, these findings indicate that mTOR regulates ribosome biogenesis at multiple levels in response to fluctuating growth conditions.

## 4 p53-dependent response to nucleolar stress

Nucleolus, while being a site and factory for ribosome biogenesis, indirectly senses stress signals and initiates multiple signaling cascades to deal with environmental challenges (Boulon et al., 2010; Golomb et al., 2014). Of particular interest are signaling pathway involving ribosomal proteins RPL11 and RPL5, as well as 5S rRNA, which plays a unique role in transmitting stress signals upon disruption of ribosome biogenesis and activation of p53 activation (Bursac et al., 2014; Golomb et al., 2014).

**FIGURE 2:**
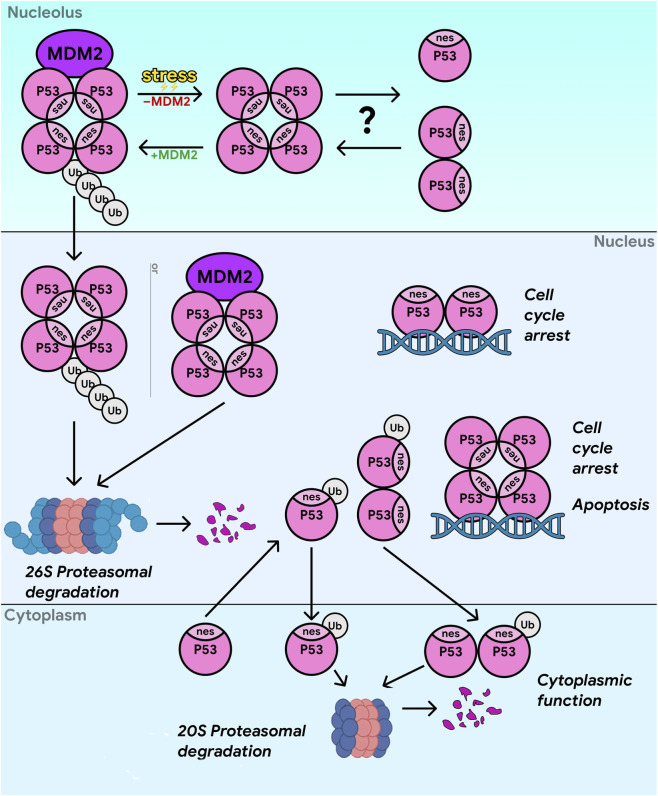
The tetramerization of p53 increases its DNA-binding affinity and transactivation by affecting transport between nucleus and nucleolus. Tetramerization is required for the ubiquitin-dependent degradation of p53 (mediated by the 26S proteasome). Dimers and monomers primarily degrade via ubiquitin-independent mechanisms (20S proteasome). The Nuclear Export Signal (NES) within the tetramerization domain (TD) of p53 controls its localization. Monomers expose the NES, which retains p53 in the cytoplasm. Tetramerization provides a masking of the NES, leading to nuclear localization. p53 oligomerization status can modulate cell fate decisions between growth, cell cycle arrest and apoptosis. The transport of p53 is dependent on ubiquitination. Monoubiquitination at low levels of MDM2 promotes the nuclear export of p53, while high levels of MDM2 promote polyubiquitination and nuclear degradation of p53. In the direct nucleolar control model, stress directly disrupts the efficient process of p53 polyubiquitination, which normally occurs within the nucleolus itself. It is possible that an oligomer/monomer transition takes place in the nucleolus, which could explain the rapid exit from the nucleolus.

p53 (encoded by TP53 gene) is a famous tumor suppressor responding to stresses such as DNA damage or impaired ribosome biogenesis by stabilization of its polypeptide, and hence, increasing concentration of active protein which level is remarkably low under normal conditions. p53 is a transcription factor controlling transcription of many genes, including p21/WAF1 (Figure 2), which is a negative regulator of the cell cycle. p21 inhibits G1phase cyclins preventing their binding to CDK (cyclin-dependent kinases) and arresting cells in G1 phase. In addition, p21 suppresses cyclin B, which is required for the G2/M transition. If DNA is damaged, p53 stimulates the synthesis of DNA repair proteins. The G1 arrest is further amplified through a positive feedback loop. DNA breaks increase level of p53 which activates its target genes (Vousden and Prives, 2009; Zilfou and Lowe, 2009; Bursac et al., 2014). Moreover, p53 inhibits both Pol I transcription by binding to the SL1, necessary for Pol I recruitment to the rDNA (Derenzini et al., 2017), and Pol III transcription by binding to TFIIIB (Felton-Edkins et al., 2003; Derenzini et al., 2017). Dysregulation of p53, observed in half of all human malignant tumors, leads to uncontrolled proliferation, genomic instability, and the evolution of stress-damaged cells, promoting their survival and malignant transformation (Levine and Oren, 2009; Zilfou and Lowe, 2009; Bursac et al., 2014). In tumor cells, where wild-type p53 is preserved, p53 functions are likely inactivated due to defects in upstream or downstream components of the p53 regulatory network (Vousden and Prives, 2009; Bursac et al., 2014).

The level of p53 is regulated post-translationally, ubiquitination of p53 is mediated by MDM2 and drives proteasomal degradation of p53 (Marchenko et al., 2007). Transcription of MDM2 gene is controlled by p53, forming a negative feedback loop releasing p53-mediated suppression of cell cycle after removal of the stress and repair of damage (Haupt et al., 1997). Stabilization of p53 requires cessation of its degradation by various means converging on the disruption of the p53–MDM2 interaction (Boyd et al., 2011). MDM2, an E3 ubiquitin ligase, possesses intrinsic enzymatic activity and can directly ubiquitinate p53. MDM4, a paralog of MDM2, lacks ubiquitin ligase activity but, by forming a heterodimer with MDM2, markedly enhances MDM2’s ability to ubiquitinate p53 (Figure 2). MDM2/MDM4 complexes suppress p53 activity and control its cellular levels using several mechanisms. By direct binding to p53, they block p53 ability to activate target genes. MDM2-mediated ubiquitination tags p53 for proteasomal degradation (Zhu et al., 2025) and regulates p53 transport. Mono-ubiquitination at low MDM2 levels promotes nuclear export of p53, which does not lead to degradation but is associated with mitochondrial translocation and apoptosis (Marchenko et al., 2007; Zhu et al., 2025). By contrast, high MDM2 levels promote polyubiquitination of p53, and its degradation in the 26S proteasome within the nucleus (Figure 2) (Boyd et al., 2011). Evidence suggests polyubiquitination of p53 occurs selectively within the nucleolus. This is supported by findings that mutations, preventing MDM2 nucleolar localization, specifically disrupt p53 polyubiquitination (Figure 2) (Vlatković et al., 2014). Both p53 and MDM2 continuously and rapidly shuttle through the nucleolus, such that nearly all p53 molecules (or at least the vast majority) reside there at some point. Nucleolar p53 is ubiquitinated with a characteristic pattern showing more polyubiquitination than in the nucleoplasm or cytoplasm (Boyd et al., 2011). Upon proteasome inhibition, polyubiquitinated p53 accumulates in nucleoli (Vlatković et al., 2014). Non-mutually exclusive models have been proposed to explain how nucleolar disruption stabilizes p53. In the relocalization model, stress triggers the relocalization of nucleolar proteins (e.g., ribosomal protein L11) into the nucleoplasm, where they bind MDM2 and inhibit its ability to ubiquitinate p53. In the direct nucleolar control model, stress directly impairs efficient polyubiquitination of p53, which normally occurs in the nucleolus, even in the absence of detectable protein relocalization (Vlatković et al., 2014). Importantly, although MDM2 is the predominant E3 ligase for p53 *in vivo*, about twenty p53-targeting ubiquitin E3 ligases have been identified to date. Some of these restrict nuclear accumulation of p53, while others facilitate its nuclear export, thereby promoting cytoplasmic retention (Rodríguez, 2014). The ability of the tumor suppressor p53 to form dimers and tetramers represents another regulatory layer of its activity, stability, and subcellular localization. In the absence of stress, p53 exists predominantly as dimers (∼59%) and monomers (∼28%), with tetramers—required for its function—being relatively scarce (∼13%) (Holoubek et al., 2025). p53 oligomerization status can modulate cell fate decisions between growth, cell cycle arrest and apoptosis (Fischer et al., 2016). Upon activation, for example after DNA damage, the proportion of functional tetramers increases sharply, reaching ∼93% of total p53. This occurs both due to overall p53 accumulation and the action of additional factors that accelerate tetramer assembly. In some stress contexts, an increase in tetramer content precedes the rise in overall p53 levels. Certain post-translational modifications, such as phosphorylation or acetylation, accelerate tetramerization. The tetrameric form of p53 binds DNA ∼1,000 times more efficiently than the monomer, making it a potent transcription factor. Some critical post-translational modifications, such as phosphorylation at Ser15—which impairs MDM2 binding—occur only after tetramerization. p53 possesses a nuclear export signal (NES) (Figure 2) (Holoubek et al., 2025). In monomers and dimers, this signal is exposed, enabling export to cytoplasm. In tetramers, the NES is masked, retaining active p53 in the nucleus. MDM2-mediated ubiquitination of p53 also depends on its oligomeric state. Tetramers are efficiently ubiquitinated by MDM2 and degraded via the ubiquitin-dependent 26S proteasome pathway (Figure 2). By contrast, monomers and dimers (including oligomerization-domain mutants) are poorly ubiquitinated by MDM2, but can be degraded by an alternative, ubiquitin-independent proteasomal pathway (Gencel-Augusto and Lozano, 2020). For example, MYBBP1A, a protein involved in rRNA transcription and processing, modulates p53 oligomerization. In the presence of ectopically expressed MYBBP1A, p53 is predominantly detected in high-molecular-weight fractions (Ono et al., 2014; Gencel-Augusto and Lozano, 2020). Interestingly, p53 dimers bind MDM2 more efficiently than monomers and undergo ubiquitin-independent degradation. It was found (Kulikov et al., 2010) that MDM2 can directly bind the proteasome, enhancing p53–proteasome association and promoting efficient degradation; however, whether MDM2 plays an active role in modulating p53 oligomerization remains an open question (Gencel-Augusto and Lozano, 2020).

It has been proposed that translocation of the p53/MDM2 complex to the cytoplasmis required for their proteasomal degradation although there are evidence that proteasome-mediated p53 degradation can also occur in the nucleus (Shirangi et al., 2002; Golomb et al., 2014). Moreover, it likely can occur even in the nucleolus in a ubiquitin-independent, calpain-dependent manner (Tao et al., 2013; Golomb et al., 2014). Activity of p53 is also dependent on post-translational modifications such as phosphorylation (Golomb et al., 2014) and acetylation (Brooks and Gu, 2011; Golomb et al., 2014) of p53 or MDM2 (Golomb et al., 2014). These modifications disrupt p53/MDM2 interaction, promote p53 tetramerization, and enhance its binding to target genes (Golomb et al., 2014). NPM1 facilitates MDM2 dephosphorylation, thereby enhancing p53 degradation (Maggi et al., 2014; Luchinat et al., 2018; González-Arzola et al., 2022). Furthermore, NPM1 directly interacts with p53, suppressing tumor cell growth, and this interaction activates p53-mediated transcription (Luchinat et al., 2018). Several observations suggested existence of a cell cycle checkpoint triggered by disruption of ribosome biogenesis (Bursac et al., 2014; Dong et al., 2020). Expression of dominant-negative mutants of Bop1 (a pre-60S biogenesis factor and member of the PeBoW complex) and several other factors inhibiting rRNA processing has been shown to prevent cell cycle progression in a p53-dependent manner. Similarly, inhibition of rRNA processing by 5-fluorouracil (Sun et al., 2007; Nishimura et al., 2015) or reduced expression of proteins required for 18S and 28S rRNA maturation—such as hUTP1 (Hölzel et al., 2005), PAK1IP1(Yu et al., 2010), WDR3 (Yu et al., 2010), WDR12 (Hölzel et al., 2005), WDR36 (Skarie and Link, 2008), nucleophosmin (NPM, B23) (Skarie and Link, 2008), nucleostemin (Table 1) (Dai et al., 2008), and multiple 40S or 60S ribosomal proteins (Table 2) (Fumagalli et al., 2009) can also induce p53-mediated stress signaling. Importantly, for most ribosome biogenesis factors, pre-rRNA processing defects caused by their depletion were p53-independent. For example, in (Tafforeau et al., 2013), pre-rRNA processing defects were examined after depletion of 21 factors in HCT116 cells in the presence or absence of p53, and the resulting phenotypes were nearly identical. However, for some factors, such as SURF6, processing defects after knockdown were p53-dependent and did not trigger p53-mediated cell cycle arrest (Moraleva et al., 2023).

**TABLE 2:** Extraribosomal functions of large and small ribosomal subunits proteins (RPL and RPS, respectively) in stress signaling pathways.

RPL	MDM2 binding ability	Participation in p53-independent response	Bibliography
RPL3	Binds and stabilizes p21 protein and downregulates MDM2	Activates p21 via Sp1, inhibits E2F1 via PARP1, reduces cyclin D1, causes cycle arrest in p53-null cells, stabilizes IκB, which inhibits nuclear transport and NF-κB activation	Russo et al. (2013), Russo et al. (2015), Pecoraro et al. (2019), Maehama et al. (2023), González-Arzola (2024)
RPL5	Forms a complex with RPL11 and 5S rRNA, inhibits the ubiquitin ligase activity of MDM2, stabilizes p53	RPL5 mutant shows p53-independent G2/M cell cycle delay at the ES stage	Bursać et al. (2012), Fumagalli et al. (2012), Donati et al. (2013), Bursac et al. (2014), Russo et al. (2015)
RPL11	A key stress mediator; binds to MDM2, inhibits p53 degradation; also binds to MYC, inhibiting its transcriptional activity	Inhibits c-MYC (binding to mRNA and protein), interacts with p14ARF to activate p21, degrades E2F-1 via MDM2	Dai et al. (2007), Challagundla et al. (2011), Donati et al. (2011), Donati et al. (2012), Maehama et al. (2023)
RPL14	Not directly listed, but mentioned as a negative regulator of c-MYC (may indirectly affect the p53/MDM2 pathway)	Not described	Rao et al. (2012)
RPL19	Not described	Not specified (but overexpression in cancer increases stress sensitivity)	Hong et al. (2014), Weeks et al. (2019)
RPL22	Not directly stated, but mentioned as a negative regulator of c-MYC	Not described	Rao et al. (2012)
RPL23	binds to MDM2, inhibits it, stabilizes p53	Not described	Bursać et al. (2012), Fumagalli et al. (2012), Bursac et al. (2014), Nishimura et al. (2015), Turi et al. (2019)
RPL24	Not directly stated, but deficiency causes p53-dependent disorders	Not described	Barkić et al. (2009)
RPL26	Binds to MDM2, inhibits p53 degradation	RPL26 can induce cell cycle arrest and apoptosis in the absence of p53 by regulating p73, a p53 family member	Ofir-Rosenfeld et al. (2008), Zhang et al. (2010), Bursać et al. (2012), Fumagalli et al. (2012), Bursac et al. (2014), Sun and Dai (2016), Micic et al. (2020)
RPL29	Not stated directly, but deficiency activates p53	Not described	Jin et al. (2004)
RPL30	Not stated directly, but deficiency activates p53	Not described	Jin et al. (2004)
RPL37	Mentioned that it can bind MDM2	Not described	Jin et al. (2004); Zhou et al. (2012a)
RPL7a	Not stated directly, but deficiency activates p53	Not described	Fumagalli et al. (2009)
RPS3	Binds to MDM2, inhibits its activity, and increases p53 stability	Not described	Yadavilli et al. (2009)
RPS6	Not directly specified, but involved in mTOR-mediated regulation of ribosome biogenesis; deficiency activates p53	Not described	Fumagalli et al. (2009)
RPS7	Binds to MDM2, inhibits p53 ubiquitination	Not described	Chen et al. (2007), Fumagalli et al. (2009), Fumagalli et al. (2012), Zhang and Lu (2009), Zhu et al. (2009), Bursać et al. (2012), Zhou et al. (2012a), Hein et al. (2013), James et al. (2014), Nishimura et al. (2015)
RPS9	Not directly stated, but deficiency activates p53	Not described	Fumagalli et al. (2009)
RPS11	Not directly stated, but increased expression in cancer (glioblastoma), prognostic marker	Not described	Yong et al. (2015), Weeks et al. (2019)
RPS14	Binds to MDM2, inhibits it, stabilizes p53; also interacts with c-MYC mRNA (together with RPL11)	Participates in p53-independent suppression of c-MYC (binding to the 3′-UTR of c-MYC mRNA)	Jin et al. (2004), Challagundla et al. (2011), Donati et al. (2012), Zhou et al. (2012a), Liao et al. (2013), Li et al. (2015), McCool et al. (2020), Maehama et al. (2023)
RPS15	Not stated directly, but deficiency activates p53	Not described	Zhou et al. (2012a), Daftuar et al. (2013)
RPS19	Not directly stated, but mutations are associated with Diamond-Blackfan anemia; deficiency activates p53	Not described	Jin et al. (2004), Narla et al. (2011), Golomb et al. (2014)
RPS20	Bind to MDM2. Also, increased expression in glioblastoma is a prognostic marker	Not described	Zhou et al. (2012a), Daftuar et al. (2013), Yong et al. (2015), Weeks et al. (2019)
RPS24	Not directly stated, but knockdown inhibits colon cancer cell migration and proliferation	Not described	Wang et al. (2015), Weeks et al. (2019)
RPS25	Binds to MDM2, inhibiting its activity	Not described	Zhang et al. (2012)
RPS27	Binds to MDM2	Not described	Xiong et al. (2011)
RPS27A	Binds to MDM2, inhibits p53 ubiquitination	Not described	Sun et al. (2011)
RPS27L	Binds to MDM2	Not described	Xiong et al. (2011)

The table compiles data on the ability of various RPLs, and RPSs, to bind the ubiquitin ligase MDM2 and thereby modulate the stability of the tumor suppressor p53, as well as their involvement in p53-independent cellular responses to ribosome biogenesis stress. For each protein, corresponding bibliographic references to sources cited in the review’s reference list are provided.

Subsequent studies showed that other disruptions in ribosome biogenesis can also trigger a p53 response (Zhang and Lu, 2009; Bursac et al., 2014). Inhibition of rRNA transcription can lead to nucleolar functional changes and p53 stabilization (Bywater et al., 2012; Bursac et al., 2014). Additionally, blocking nuclear import or export of ribosomal subunits disrupts ribosome biogenesis and activates p53 response (Daftuar et al., 2013; Bursac et al., 2014). Taken together, these findings suggest that inhibiting ribosome biogenesis at various levels often leads to subsequent p53 accumulation.

Ribosomal proteins RPL5 and RPL11 are directly involved in activation of p53. Early studies showed that RPL5 forms an extraribosomal complex with MDM2, p53, and 5S rRNA (Marechal et al., 1994; Bursac et al., 2014). Later works demonstrated that R,PL5 and RPL11 bind MDM2, blocking its E3 ligase function and promoting p53 accumulation (Dai et al., 2004). Depletion of RPL5 or RPL11is sufficient to suppress p53 activation upon ribosome biogenesis inhibition at various levels, as these proteins are interdependently essential for this response (Bursać et al., 2012; Fumagalli et al., 2012; Zhou et al., 2012b; Bursac et al., 2014).

The interaction of RPL5 and RPL11 with MDM2 is not unique, as ribosomal proteins—such as RPL23 (Nishimura et al., 2015; Turi et al., 2019), RPL26 (Ofir-Rosenfeld et al., 2008; Zhang et al., 2010; Micic et al., 2020) and others (Table 2), when overexpressed in cells bind MDM2 and inhibit its ubiquitin ligase activity thereby upregulating p53 (Warner, 1977; Bursac et al., 2014).

Interestingly, depletion of RPL5 or RPL11 inhibits ribosome biogenesis (Fumagalli et al., 2012; Donati et al., 2013; Bursac et al., 2014) but does not trigger a p53 response. This suggests that RPL5 and RPL11 are important transducers of p53 activation signals during ribosome biogenesis stress (Zhang and Lu, 2009; Deisenroth and Zhang, 2010; Bursać et al., 2012; Fumagalli et al., 2012; Bursac et al., 2014). In contrast, ribosome biogenesis inhibition caused by depletion of RPL23, RPL26, or RPS7— as well as depletion of other ribosomal proteins except RPL11 and RPL5—activates p53 in an RPL5/RPL11-dependent manner (Bursać et al., 2012; Fumagalli et al., 2012; Bursac et al., 2014). It has been proposed that depletion of specific ribosomal proteins may reduce ribosome numbers, and their inhibitory effect on p53 accumulation may be explained by global translational suppression rather than loss of their specific effects on MDM2 function (Fumagalli et al., 2012; Bursac et al., 2014). Alternative hypothesis suggests that a number of ribosomal proteins, including RPL5, RPL11, RPL23, RPL26, and RPS7, passively diffuse from the nucleolus into the nucleoplasm, where they bind MDM2 and inhibit its anti-p53 activity (Deisenroth and Zhang, 2010; Bursać et al., 2012; Zhou et al., 2012a; Bursac et al., 2014). However, nucleolar disruption and passive diffusion of ribosomal proteins do not necessarily occur during ribosome stress; but may result in increased translation of RPL5 and RPL11 mRNA.

Excessive production and nuclear import of ribosomal proteins may be sufficient for p53 activation (Donati et al., 2011; Bursac et al., 2014). In particular, impaired 40S ribosome biogenesis increases RPL11 mRNA transcription and does not significantly affects 60S biogenesis or nucleolar integrity (Nishimura et al., 2015). Excessive RPL11 presumably translocates to the nucleus, where it interacts with MDM2, and blocks its function, resulting in p53 stabilization. In contrast, inhibition of 60S biogenesis impairs RPL11 mRNA translation (Nishimura et al., 2015; Zhou et al., 2015). Most ribosomal proteins are synthesized during ribosome biogenesis inhibition but undergo ubiquitin-independent proteasomal degradation to prevent toxic accumulation of free ribosomal proteins (Andersen et al., 2005; Bursać et al., 2012; Bursac et al., 2014). Conversely, RPL5, RPL11, and 5S rRNA are redirected from 60S ribosome assembly to MDM2 inhibition in the cytoplasm and nucleoplasm during ribosome stress (Bursać et al., 2012; Donati et al., 2013; Bursac et al., 2014). An additional complication is the existence of a homologue of MDM2, namely MDMX (also known as HDMX/MDM4/HDM4), which works in concert with MDM2 to degrade p53 (Klein et al., 2021). Thus, 5S rRNA may act as a positive or negative regulator of p53, depending on its association with the RPL5-RPL11-MDM2 complex or MDMX, respectively. In this context, it is possible that the L11-MDM2-p53 signaling pathway may mediate a p53-dependent checkpoint in response to deregulated oncogenes that promote excessive ribosome biogenesis (Macias et al., 2010).

## 5 p53-ARF axis restricts ribosome biogenesis

**FIGURE 3:**
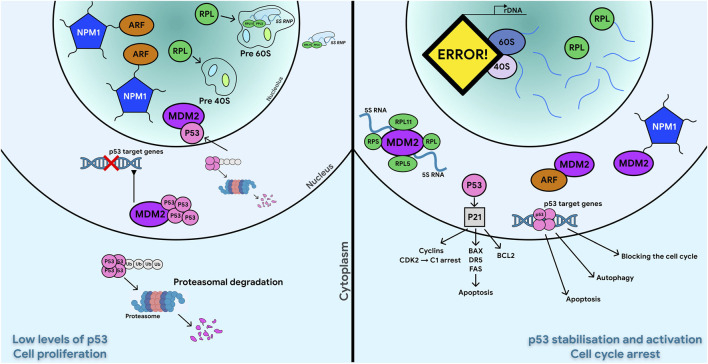
Small (40S) and Large (60S) subunit ribosomal proteins (RPs) promote accumulation of functional p53 and ARF and induce cell cycle arrest under nucleolar stress conditions. Normal conditions are shown on the left, while stress-induced changes–on the right. Under normal growth conditions, p53 is targeted by the E3 ubiquitin ligase MDM2 for degradation, keeping p53 at low levels. During nucleolar stress, ribosome biogenesis is inhibited, and free ribosomal proteins (RPL and RPS) accumulate in the nucleoplasm. Specifically, rpL5-rpL11-5srRNA form a complex that interacts with MDM2. This binding stabilizes p53, which in turn promotes the transcription of its downstream targets. In unstressed cells, ARF is associated with NPM1 in the nucleoli, allowing MDM2 targeting of p53 and causing its nuclear export and proteasomal degradation, which keeps p53 at low level. In response to nucleolar stress, NPM1 and ARF are released in the nucleoplasm where they bind to MDM2, thus preventing the proteasomal degradation of p53.

**FIGURE 4:**
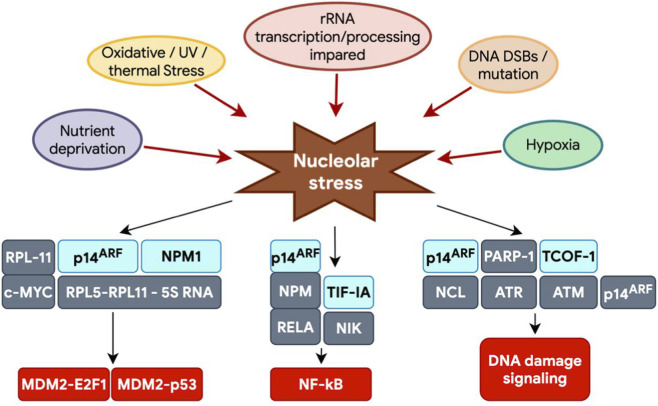
Schematic representation of c-Myc and p53 functions controlled by NPM and ARF. The MDM2-p53 duo is considered a central hub controlling p53-mediated antitumor activity. Mechanisms that disrupt the MDM2-p53 interaction lead to p53 activation, which in turn induces MDM2 expression through negative feedback. ARF promotes p53 activation by inhibiting MDM2. Loss of p53 often leads to ARF induction. Both mTOR and c-Myc induce p53 activation upon initial exposure to DNA damage stress, but both can promote tumorigenesis by inhibiting p53 activity.

Interaction between MDM2 and p53 could be prevented by binding of modulator proteins to MDM2, directly or by sequestering MDM2 in a different cellular compartment (Weber et al., 1999; Golomb et al., 2014). The best-known example of this modulators is the tumor suppressor ARF (p14ARF in humans, p19ARF in mice). This protein accumulates under excessive mitogenic signaling, such as c-MYC overexpression, and induces p53-dependent apoptosis or growth arrest (Lessard et al., 2010; Golomb et al., 2014). The primary mechanism of ARF action is MDM2 inhibition, which prevents p53 degradation and enhances its antitumor activity (Kung and Weber, 2022) in response to a broad range of oncogenic stimuli, including increased expression of MYC, E2F1, RAS, E1A, and v-Abl (Macias et al., 2010). Mutations in ARF are found in about 40% of tumors (Lessard et al., 2010). Transgenic mice lacking ARF spontaneously developed tumors over 1 year, which include sarcomas, lymphomas, carcinomas, and nervous system tumors (Lahav et al., 2004; Kung and Weber, 2022). It has been shown that ARF can inhibit the cell cycle even in the absence of p53 mediating p53-independent tumor suppression (Figures 3, 4) (Rocha et al., 2003; Kelly-Spratt et al., 2004; Sandoval et al., 2004; Korgaonkar et al., 2005; Muniz et al., 2011; Forys et al., 2014; Kung and Weber, 2022). ARF is encoded by the CDKN2A locus, which also encodes another tumor suppressor, p16INK4A. ARF and p16INK4A are transcribed from two partially overlapping open reading frames and translated into two unrelated proteins. Two arginine-rich domains (amino acids 1–14 and 82–101) of ARF mediate its nucleolar localization (Kung and Weber, 2022). The N-terminal motif (amino acids 1–14) of ARF interacts with the central domain of MDM2 inhibiting its ubiquitin ligase activity (Figures 3, 4) (Lohrum et al., 2000; Weber et al., 2000; Kung and Weber, 2022). ARF inhibits MDM2 by preventing its translocation from the nucleoplasm to the nucleolus (Figure 3) (Lin and Lowe, 2001; Llanos et al., 2001; Korgaonkar et al., 2005). Notably, even non-nucleolar forms of ARF can activate p53 and suppress proliferation (Korgaonkar et al., 2005), indicating that nucleolar localization of ARF is not essential for its function. Besides MDM2, ARF binds numerous other proteins, sequestering them in the nucleolus upon overexpression (Korgaonkar et al., 2005). The interaction between ARF and NPM1 in the nucleolus increases ARF stability and functional activity (Figures 3, 4) (Pandit and Gartel, 2011; Maggi et al., 2014; Luchinat et al., 2018). NPM1 protects ARF from proteasomal degradation by retaining it in the nucleolus, and also controls ARF availability (Quin et al., 2014; González-Arzola et al., 2022; Riback et al., 2023). Most cellular ARF appears bound to NPM1, whereas only a small fraction of NPM1 associates with ARF. Under normal conditions, this interaction maintains a balance allowing ARF to persist in cells and remain inactive. Upon cellular stress such as DNA damage or oncogenic activation, ARF is released from the NPM1 complex (Lee et al., 2005; González-Arzola et al., 2022), enabling its exit to the nucleoplasm interaction with MDM2, blockage of MDM2 ubiquitin ligase activity and stabilization of p53 (Figures 3, 4) (Rubbi and Milner, 2003). Knockdown of NPM1 specifically reduces nucleolar localization of ARF, significantly enhances the association of ARF with human MDM2, and activates p53, while NPM1 overexpression counteracts ARF function by increasing ARF nucleolar retention. In acute myeloid leukemia associated with NPM1 mutations, this mechanism is disrupted. Overexpression or mutant forms of NPM1 causes abnormal sequestration of ARF in the nucleolus, preventing its interaction with MDM2 and subsequent p53 activation (Colombo et al., 2006; Moulin et al., 2008; Kung and Weber, 2022). Interestingly, the ARF-NPM1 relationship constitutes a complex negative feedback system (Figure 4). While NPM1 regulates ARF localization and stability, ARF itself can affect NPM1 expression by reducing its stability and function, thereby impairing its role in rRNA processing (Korgaonkar et al., 2005; González-Arzola et al., 2022; Kung and Weber, 2022). This mutual regulation creates a delicate balance controlling proliferation of normal cells and contributing to tumor growth, when disrupted. Notably, even under physiological conditions, such as in aging cells, most ARF remains in complex with NPM1, highlighting the importance of this interaction for cellular homeostasis (Bertwistle et al., 2004; Korgaonkar et al., 2005). The ARF-NPM interaction is also sensitive to stress factors, including AKT, cytochrome c, and CD24 (Brady et al., 2004; Hamilton et al., 2014; Kung and Weber, 2022). As a nucleolar protein, ARF suppresses tumor growth by disrupting ribosome biogenesis, rRNA processing (Sugimoto et al., 2003; Lessard et al., 2010), and its translation (Cottrell et al., 2020; Kung and Weber, 2022). ARF regulates these processes through multiple mechanisms: direct interaction with the rRNA promoter, hindrance of RNA polymerase I transcription termination factor (TTF-I) import into the nucleolus (Colombo et al., 2005; Lessard et al., 2010), inactivation of UBF (Ayrault et al., 2006), suppression of the rRNA processing enzyme Drosha, and modulation of nucleolar localization of the RNA helicase DDX5 (Saporita et al., 2011; Kuchenreuther and Weber, 2014; Kung and Weber, 2022). Notably, ARF’s ability to interact with DDX5 also prevents DDX5-c-MYC interaction, disrupting a positive feedback loop that enhances c-MYC-mediated transcription (Sugimoto et al., 2003; Kung and Weber, 2022). Among many regulators, mTOR (mechanistic target of rapamycin) and the oncogene c-MYC affect ARF, MDM2 and p53 levels and activity, controlling malignant transformation processes (Figure 4) (Maggi et al., 2014; Hafner et al., 2019; Klein et al., 2021; Kung and Weber, 2022) (Figures 3, 4). Under cellular stress, p53 suppresses mTOR activity in two ways: via activation of the TSC1/TSC2 complex (mTOR inhibitor) involving AMPK kinase and sestrin-1/2 proteins, or by stimulating PTEN synthesis, which blocks AKT—an mTOR activator (Feng et al., 2005; Budanov and Karin, 2008; Kung and Weber, 2022). Conversely, oncogenic activation enhances mTOR-mediated translation of ARF mRNA boosting p53 activity and suppressing tumor growth. Additionally, mTOR can activate p53 through the kinase S6K1, which phosphorylates MDM2, impairing its nucleolar localization and thus stabilizing p53 (Lee et al., 2007; Lai et al., 2010; Kung and Weber, 2022). Therefore, mTOR and p53 mutually regulate each other, playing a key role in tumor suppression. c-MYC induces ARF expression and p53-dependent apoptotic programs in the initial DNA damage response but ultimately leads to inactivation of the ARF-MDM2-p53 pathway (Nieminen et al., 2013; Phesse et al., 2014; Kung and Weber, 2022). To suppress c-MYC-driven tumorigenesis, p53 can transcriptionally repress c-MYC directly by promoting histone deacetylation or indirectly via induction of microRNA (miR)-145 (Ho et al., 2005; Sachdeva et al., 2009; Kung and Weber, 2022). ARF directly interacts with c-MYC or its transcriptional cofactor Miz1 to induce growth arrest and cell death even in the absence of p53 (Herkert et al., 2010; Kung and Weber, 2022). Two parallel MDM2-mediated pathways maintain p53 activity to counteract oncogenic functions of c-MYC. Besides ARF-MDM2 interaction, ribosomal proteins interacting with MDM2 are also required to maximize p53 activity in inhibiting c-MYC-driven tumorigenesis (Macias et al., 2010; Kung and Weber, 2022). Recently, c-MYC was shown to regulate the p53-MDM2-ARF tumor suppression axis by modulating two different long noncoding RNAs (Jain, 2020; Xu et al., 2020; Kung and Weber, 2022). One, SENEBLOC, acts as a scaffold promoting p53-MDM2 association leading to p53 degradation. The other, MILIP, inhibits SUMOylation, reducing p53 SUMO modification (Kung and Weber, 2022). SUMOylation of p53 is an important post-translational modification mechanism by which MDM2 and ARF regulate p53 functions (Kung and Weber, 2022). ARF also mediates SUMOylation of NPM and MDM2 (Tago et al., 2005; Kung and Weber, 2022). c-MYC expression strongly elevates p53 level in ARF-null MEF cells. Given c-MYC’s role in stimulating ribosome biogenesis, this ARF-independent induction of p53 by c-MYC is likely mediated by ribosomal proteins. This suggests that p53 protects against c-MYC oncogenesis through two independent signaling pathways: ARF-MDM2-p53 and RP-MDM2-p53. Accordingly, disruption of either accelerates c-MYC-induced tumorigenesis (Macias et al., 2010). Data confirm the RPL11-MDM2-p53 pathway is an important *in vivo* barrier against c-MYC-driven oncogenesis, alongside ARF-MDM2-p53 signaling. Together, these results highlight the complexity of ribosome biogenesis regulation via the p53-ARF axis, suggesting this tumor suppressive pathway is a crucial barrier to tumorigenesis.

The study of MDM inhibitors has become a promising direction in cancer therapy, with numerous candidates reaching clinical trials. Nutlin-3a and RG7388 (Idasanutlin) are MDM2-p53 interaction inhibitors. Unlike direct Pol I inhibitors, Nutlin-3a and RG7388 aim to restore p53 function in tumors carrying wild-type *TP53*. They bind to the p53-binding pocket of the MDM2, preventing p53 degradation and leading to its stabilization and activation (Zhu et al., 2025). Nutlin-3a was the first compound, demonstrating preclinical efficacy, but its clinical application is hindered by low bioavailability, rapid systemic clearance, and the development of resistance, often associated with acquired *TP53* mutations (Zhu et al., 2025). RG7388 is next-generation compound with nanomolar activity and increased selectivity. It showed promising results in early clinical trials. However, a large Phase III trial (MIRROS) evaluating its combination with cytarabine for acute myeloid leukemia did not show an improvement in overall survival compared to cytarabine alone. Furthermore, treatment was often accompanied by significant gastrointestinal and hematological toxicities, highlighting challenges with the therapeutic window (Zhu et al., 2025).

## 6 p53-independent pathways of nucleolar stress response

The existence of a p53-independent pathway regulating the interconnection between ribosome biogenesis and cell proliferation in mammals was first postulated based on the observation that selective inhibition of rRNA synthesis by actinomycin D could disrupt cell cycle progression even in p53-depleted cells, although to a lesser extent than in p53-proficient cells (Donati et al., 2012; Maehama et al., 2023). An example is the depletion of the Pes1 protein. Knockdown of Pes1—a nucleolar protein that is part of the PeBoW complex with Bop1 and WDR12 and is involved in pre-60S ribosome biogenesis—caused defects in ribosome biogenesis, stabilized p53, and led to cell cycle arrest (Grimm et al., 2006; Donati et al., 2012). However, Pes1 knockdown also impaired proliferation in p53-deficient cells, indicating the presence of p53-independent cell cycle control in these cells (Li et al., 2009; Donati et al., 2012). The underlying regulatory mechanism was associated with downregulation of the cell cycle protein cyclin D1 and upregulation of the CDK inhibitor p27, which plays a critical role in controlling the G1/S transition, leading to significant cell cycle slowdown (Montanaro et al., 2007; Morishita et al., 2008; Iadevaia et al., 2010).

One mechanism for stabilizing p27 in p53-deficient cells involves nucleolar stress-induced destabilization of the proto-oncogenic serine/threonine kinase PIM1 (Iadevaia et al., 2010; Olausson et al., 2012). Under normal conditions, PIM1 phosphorylates p27, targeting it for subsequent degradation via the ubiquitin-proteasome pathway. Disruption of ribosome biogenesis leads to a reduction in the level or activity of PIM1 kinase. The decrease in PIM1-mediated p27 phosphorylation, in turn, prevents its proteasomal degradation, resulting in the accumulation of stable p27 protein in the cell (Iadevaia et al., 2010; González-Arzola, 2024).

Other key players in p53-independent pathways are E2F-1, MYC, NFκB, and ribosomal proteins (Maehama et al., 2023). E2F-1 is responsible for activating the transcription of a broad spectrum of genes necessary for the G1/S transition of the cell cycle (Pfister et al., 2015; Pecoraro et al., 2019). A central mechanism for regulating E2F activity is its binding to the retinoblastoma protein (pRB), which occurs in a cell cycle-dependent manner. It has been shown that NPM1 participates in transporting the hyperphosphorylated form of pRB into the nucleolus. This process facilitates the dissociation of the pRB-E2F complex, releasing the active E2F factor, which then initiates the transcription of genes required for DNA synthesis (Takemura et al., 2002; Taha and Ahmadian, 2024). Under normal conditions, the stability of one of the key members of this family, E2F-1, is maintained through its interaction with the ubiquitin ligase MDM2. This interaction protects E2F-1 from proteasomal degradation by preventing the binding of other E3 ubiquitin ligases responsible for its polyubiquitination (Iadevaia et al., 2010; Donati et al., 2012). Upon disruption of ribosome biogenesis, the ribosomal protein RPL11 is released and migrates from the nucleolus to the nucleoplasm. RPL11 specifically binds to MDM2, blocking its stabilizing function towards E2F-1 (Donati et al., 2011; 2012). This, in turn, promotes MDM2-mediated polyubiquitination of E2F-1 and its subsequent degradation by the proteasome. Importantly, unlike the classical p53-dependent stress response where RPL11 binding to MDM2 stabilizes p53, in the case of E2F-1, the same interaction accelerates its degradation (Maehama et al., 2023).

RPL11, along with other ribosomal proteins such as RPL5 and RPS14, is involved in p53-independent cell cycle arrest, directly interacting with c-MYC mRNA by binding to its 3′-UTR and suppressing translational (Challagundla et al., 2011; Donati et al., 2012; Maehama et al., 2023). Concurrently, RPL11 can bind to the c-MYC protein itself, inhibiting its transcriptional activity (Dai et al., 2007; Olausson et al., 2012). It is important to emphasize that this suppression of c-MYC activity is also observed in cells lacking functional p53, defining it as a p53-independent mechanism for controlling proliferation (Dai et al., 2007).

RPL3, large ribosomal subunit protein, also mediates p53-independent pathways. Like other ribosomal proteins, RPL3 translocates from the nucleolus to the nucleoplasm in response to stress, including genotoxic stress (Pecoraro et al., 2019; González-Arzola, 2024). A central target of RPL3’s extraribosomal function is the potent CDK inhibitor p21 (CDKN1A). RPL3 activates p21 expression at multiple levels by directly binding to transcription factor Sp1 and increasing transcription of the CDKN1A gene (Maehama et al., 2023), and by stabilizing the p21 protein (Maehama et al., 2023). This combination of effects results in p21-dependent cell cycle arrest in p53-null cells in response to disruption of ribosome biogenesis (Russo et al., 2013; González-Arzola, 2024). In nucleoplasm, RPL3 binds to poly-ADP-ribose polymerase 1 (PARP-1). Preventing PARP-1 binding to the E2F1 gene promoter, thereby blocking its transcription (Pecoraro et al., 2019; González-Arzola, 2024). Furthermore, RPL3 negatively regulates cyclin D1 by reducing its mRNA and protein levels (Pecoraro et al., 2019; González-Arzola, 2024). The biological significance of the RPL3-mediated pathway is confirmed by its role in therapy response. Notably, RPL3 expression is decreased in colorectal cancer cells, which is associated with reduced efficacy of some chemotherapeutic agents (González-Arzola, 2024). Conversely, RPL3 enhances the activity of antitumor drugs, exhibiting pro-apoptotic and anti-autophagic effects, especially in p53-deficient cancer cells (Pecoraro et al., 2020; González-Arzola, 2024). These data highlight the potential of the RPL3 pathway as a target for therapeutic modulation.

Some ribosome biogenesis factors can regulate the cell cycle through both p53-dependent and p53-independent pathways. For example, the SURF6–Rrp15–PPAN (Table 1) complex is involved in pre-60S ribosome maturation and cell cycle regulation (Broeck and Klinge, 2023; Vanden Broeck and Klinge, 2024). Depletion of Rrp15 in human and mouse cells causes arrest at the G1/S checkpoint and increases p21 expression (Wu et al., 2016). In p53-deficient cells, Rrp15 knockdown induced a metabolic shift from glycolysis to mitochondrial oxidative phosphorylation and the accumulation of reactive oxygen species (ROS) triggering cell death. Similarly, depletion of the PPAN protein inhibited apoptosis in a p53-independent manner by stabilizing BAX and inducing mitochondrial depolarization (Pfister et al., 2015).

Thus, accumulating data demonstrate that ribosome stress impedes the proliferation of mammalian cells both in the presence and absence of p53. Given that many cancers are characterized by the absence of functional p53, understanding these p53-independent pathways is of critical importance (James et al., 2014; D’Orazi, 2023). p53 stabilization in response to ribosome stress plays a key role in preventing neoplastic transformation (Macias et al., 2010; Donati et al., 2012). Disrupted ribosome biogenesis may underlie defects in p53 activation pathways and contribute to tumor development (Montanaro et al., 2012).

## 7 Integration of DNA damage, proteotoxicity and cell fate decisions via dynamic protein relocation

### 7.1 ATM and ATR and inhibition of Pol I transcription

**FIGURE 5:**
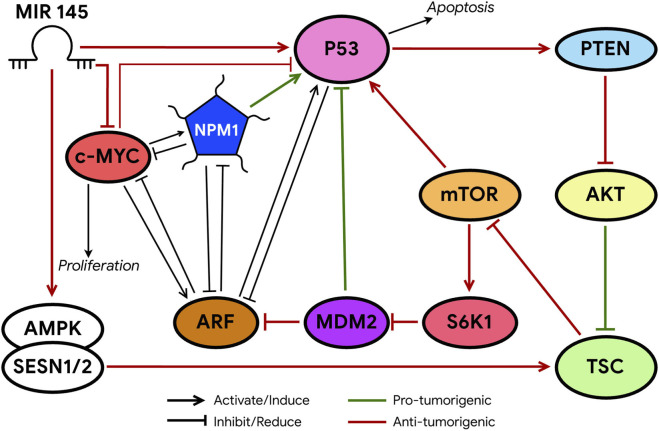
The role of nucleolar stress in transducing stress-related pathways. Nucleolar stress is initiated by DNA double-strand breaks, nutrient deficiency, hypoxia, oxidative stress, thermal stress other stress conditions. Nucleolar stress is characterized by exit from nucleolus or re-distribution of key nucleolar proteins (for example, nucleophosmin) and changes in nucleolar morphology (occur to isolate unfolded or damaged proteins in an attempt to save them using chaperones). When activated, nucleolar stress transduces damage-induced signals by dynamically controlling the nucleocytoplasmic distribution of effector molecules (p14ARF. ATM, ATR and others) involved in control of cell cycle (p53-MDM2), transcription (E2F1, NF-kB) and apoptosis (p53, p14RF). By sensing stresses on the level of nucleolus where ribosome biogenesis, control of chromatin stability, metabolic activity and induction of apoptosis interconnect, allow cells regulating the output of intersecting stress signaling cascades.

*Nucleolar* stress (for definition, please, see Introduction) has been demonstrated to serve as a sensory hub for a broad spectrum of stress stimuli, including DNA double-strand breaks (DSBs), hypoxia, nutrient deprivation, oxidative stress, and thermal stress (Russo et al., 2016; Hua et al., 2022). This process is based on the dynamic shuttling of nucleolar proteins, such as NPM1, between various cellular compartments (Hua et al., 2022). Under homeostatic conditions, their constant circulation coordinates functions both inside and outside the nucleolus. However, under stress conditions, abnormal release of resident nucleolar proteins and simultaneous sequestration of non-specifically associated proteins within the nucleolus occur (Figure 5). Emerging research has identified the activation of the NF-κB pathway as an alternative key mechanism through which nucleolar stress regulates cell growth and death (Figure 5).

Nuclear factor kappa-light-chain-enhancer of activated B cells (NF-κB) is a family of highly conserved, inducible transcription factors that factor in the stress responses and maintenance of cellular homeostasis (Gilmore, 2006; Chen and Stark, 2019). In the absence of stress, the primary NF-κB complex (RelA/p65 heterodimer) is retained in the cytoplasm by the inhibitory protein nuclear factor of kappa light polypeptide gene enhancer in B-cells inhibitor alpha (IκBα) (Gilmore, 2006; Chen and Stark, 2019). Under stress conditions (inflammation, DNA damage, nutrient starvation, etc.), IκBα is degraded, allowing NF-κB translocation to the nucleus and activation over a hundred target genes controlling proliferation, inflammation, and apoptosis (Chen and Stark, 2019). Disruption of nucleolar function (nucleolar stress)—for example, upon depletion of key nucleolar proteins such as NPM1 (Chen and Stark, 2019) or the RNA polymerase I transcription initiation factor TIF-IA (Gilmore, 2006; Chen and Stark, 2019)—also leads to NF-κB activation. This occurs via degradation of IκBα and nuclear translocation of RelA. This activation is independent of simple suppression of rRNA synthesis, indicating specific signaling mechanisms linking nucleolar integrity to the NF-κB pathway. In addition to its activation, the nucleolus can serve as a site for NF-κB sequestration. In response to specific stresses (UV irradiation, aspirin, starvation), the RelA subunit not only translocates to the nucleus but specifically accumulates in the nucleolus by interacting with p14ARF (Gilmore, 2006; Chen and Stark, 2019) and other proteins suppressesing conventional transcription. Upon entering the nucleolus, RelA induces the translocation of the multifunctional protein NPM1 from the nucleolus to the cytoplasm. In the cytoplasm, NPM1 binds the pro-apoptotic protein Bcl-2-associated X protein (BAX) and delivers it to mitochondria. This triggers the mitochondrial apoptotic pathway (Thompson et al., 2008; Khandelwal et al., 2011; Wang Z. et al., 2013; Chen and Stark, 2019). Also, NPM1 can inhibit apoptosis and co-activate NF-κB transcription, promoting expression of pro-survival genes (Lin et al., 2017; Chen and Stark, 2019; Wang et al., 2019). Existing feedback loop includes RelA changing localization and function of NPM1, and NPM1, which modulates NF-κB activity.

There is evidence that the nucleolus could be a sensor of genomic DNA stability and integrity (Hua et al., 2022). DNA double-strand breaks (DSBs) and other forms of damaged DNA can slow down or halt the Pol I transcriptional machineryinducing abnormal nucleolar dynamics and nucleolar stress (Weeks et al., 2019; Hua et al., 2022). Both functional and proteomic studies have revealed a distinct nucleolar localization for DNA repair proteins (Ogawa and Baserga, 2017; Hua et al., 2022). For instance, about 40% of poly-ADP-ribose polymerase-1 (PARP1) is stored within the nucleolus, and following DNA damage, this pool of PARP1 is activated and released into the nucleoplasm (Rancourt and Satoh, 2009; Hua et al., 2022). Two most abundant nucleolar proteins, NPM and NCL, retain repair factors within the nucleolus (Scott and Oeffinger, 2016; González-Arzola, 2024). NPM and NCL exit the nucleolus upon DSB induction (Koike et al., 2010; Kobayashi et al., 2012), with NPM translocating to DNA repair foci where it co-localizes with HR (homologous recombination) factors (breast cancer type 1 susceptibility protein (BRCA1) and radiation sensitive protein 51 (RAD50)) (Koike et al., 2010; González-Arzola, 2024).

rDNA represents one of the most intensively transcribed chromatin regions in proliferating cells (Smirnov et al., 2021; Hua et al., 2022). Сells developed specific mechanisms for repairing damaged rDNA. Specialized nucleolar DNA Damage Response (nDDR) (Velichko et al., 2021; Imamura and Yasuhara, 2025) involves rapid local suppression of rRNA synthesis mediated by the kinases Ataxia Telangiectasia Mutated (ATM) and Ataxia Telangiectasia and Rad3-related protein (ATR) (Figure 5) (Kruhlak et al., 2007; Weeks et al., 2019; Li et al., 2022; Imamura and Yasuhara, 2025). Unique initiation mechanism of signaling cascade activated by ATM is driven by in the absence of the 5′-end resection characteristic of DSB repair (Imamura and Yasuhara, 2025). Activated ATM phosphorylates the nucleolar protein TCOF1/Treacle (Ciccia et al., 2014; Imamura and Yasuhara, 2025), which serves as a key signal for recruitment complex of the meiotic recombination 11 homolog 1 (MRE11) and the DNA repair protein RAD50 so-called MRN complex to the nucleolus via the Nijmegen breakage syndrome 1 (NBS1) protein (Ciccia et al., 2014; Larsen et al., 2014; Velichko et al., 2021; Imamura and Yasuhara, 2025). This displaces Pol I from damaged loci and suppresses rRNA transcription (Kruhlak et al., 2007; González-Arzola, 2024). However, for complete transcriptional repression and subsequent efficient repair, activation of the kinase ATR is required, and it also requires TCOF1 (Mooser et al., 2020; González-Arzola, 2024). ATR enhances transcriptional repression and initiates the migration of damaged rDNA clusters to the nucleolar periphery, forming specialized structures known as “nucleolar caps” (Korsholm et al., 2019; Mooser et al., 2020; Imamura and Yasuhara, 2025). Nucleolar caps concentrate factors involved in non-homologous end joining (NHEJ) and HR (Kruhlak et al., 2007; van Sluis and McStay, 2015; Li et al., 2022), and reducing the risk of rDNA hyper-recombination (Harding et al., 2015; van Sluis and McStay, 2015; Pelletier et al., 2018). Furthermore, this mechanism physically separates homologous sequences from different chromosomes minimizing the risk of interchromosomal recombination. Thus, the nucleolus remodels and reorganizes its architecture to ensure accurate HR-mediated repair. Concurrently with kinase activation, dynamic redistribution of key nucleolar proteins, NPM1 and nucleolin (NCL), occurs. These proteins normally retain repair factors within the nucleolus. An important component of this network is also the protein p14ARF, which in response to genotoxic stress can activate the ATM/ATR pathways (Pelletier et al., 2018). Notably, mutations in the *TCOF1* gene are the cause of the ribosomopathy Treacher Collins syndrome (Ciccia et al., 2014; Larsen et al., 2014; Pelletier et al., 2018).

### 7.2 Pol I inhibitors as candidate anti-cancer therapeutics

ATM deficiency leads to impaired p53 stabilization upon DNA damage (Jankovic et al., 2013). p53 is a target of ATM, and p53 stabilization occurs, in part, through ATM phosphorylation of Ser15 in p53. ATM also phosphorylates MDM2, which plays a key role in ATM-dependent p53 stabilization. Mice expressing a form of MDM2, which cannot be phosphorylated, exhibited severe defects in apoptosis, which were even more pronounced than those observed in ATM-deficient animals and comparable to those in p53-deficient animals (Gannon et al., 2012; Jankovic et al., 2013). The development of selective small-molecule inhibitors targeting RNA Pol I transcription represents a novel therapeutic paradigm aimed at the hypertrophied ribosome biogenesis in cancer cells. Among the most studied compounds in this group are CX-5461 (pidnarulex) and BMH-21. CX-5461 (Pidnarulex) is a naphthyridine derivative that selectively inhibits Pol I transcription initiation by preventing the assembly of the pre-initiation complex, specifically by blocking the binding of the SL1/TIF-IB factor to the rDNA promoter (Drygin et al., 2011; Bywater et al., 2012; Quin et al., 2016). Its initial antitumor effect in lymphoma models, such as Eµ-Myc, was linked to nucleolar stress-induced, p53-dependent apoptosis (Bywater et al., 2012; Quin et al., 2016; Weeks et al., 2019). However, in solid tumors and p53-deficient cells, CX-5461 exhibits p53-independent activity, leading to cell cycle arrest (including G1, S, and G2 checkpoints), senescence, autophagy, or apoptosis (Drygin et al., 2011; Quin et al., 2016). A key mechanism of p53-independent action of CX-5461 is the induction of a non-canonical DNA damage response (DDR) via the activation of ATM and ATR. This occurs not due to accumulation of double-strand breaks in DNA, but as a consequence of specific disruption to rDNA chromatin structure. Blocking Pol I loading on rDNA results in the formation of “naked,” polymerase-depleted rDNA repeats, which directly triggers intra-nucleolar ATM/ATR signaling (Quin et al., 2016). The combination of CX-5461 with ATM/ATR inhibitors synergistically enhances the therapeutic effect, inducing arrest in mitosis in p53-null cells (Quin et al., 2016). CX-5461 has shown good tolerability in Phase I clinical trials and has advanced to Phase I/II trials for hematological malignancies and BRCA-deficient tumors (Pelletier et al., 2018; Zisi et al., 2022).

### 7.3 BMH-21 is an acridine derivative initially identified in a screen for p53 activators

Unlike CX-5461, its primary mechanism involves inhibiting Pol I transcription elongation by intercalating into GC-rich rDNA sequences. A distinctive feature of BMH-21 is its ability to trigger proteasomal degradation of the catalytic subunit of Pol I—POLR1A (RPA194)—leading to sustained suppression of ribosome biogenesis (Zisi et al., 2022; Corman et al., 2023). Importantly, BMH-21 induces classical nucleolar stress and p53 activation but does not cause significant DNA damage (Weeks et al., 2019; Zisi et al., 2022). Its cytotoxicity is only partially dependent on p53 and the RPL11 pathway. The drug demonstrates broad antitumor activity *in vitro* and *in vivo*, but its further development and transition to the clinic are hampered by the need to clarify the specificity of its mechanism and potential extranucleolar effects at high concentrations.

### 7.4 Proteotoxic stress response

The nucleolus plays a critically important role in protecting the cell from proteotoxic stress, particularly during heat shock (Frottin et al., 2019; González-Arzola, 2024). Although folded proteins typically enter the nucleus, the nuclear proteome contains many metastable proteins requiring quality control. Under conditions of proteotoxic stress such as heat shock, metastable and denatured nuclear proteins, along with 70 kDa heat shock proteins (Hsp70) and other molecular chaperones, are actively sequestered into the liquid-like phase of the nucleolar GC (Frottin et al., 2019). This process is mediated by interaction with resident nucleolar proteins, including NPM1 and NCL, which reduces motility of the proteins entering the nucleolus and prevents their irreversible aggregation in the nucleoplasm (Frottin et al., 2019). The molecular mechanism underlying this sequestration is LLPSdiscussed earlier (González-Arzola, 2024). Within the GC, denatured proteins stay amenable to refolding by Hsp70. It is that the proteostatic capacity of the nucleolus is limited. Prolonged stress or accumulation of pathogenic proteins associated with neurodegenerative diseases (e.g., R-dipeptide repeat-rich peptides) leads to a phase transition of the GC from a liquid-like to a solid-like, aggregated state (Frottin et al., 2019; Maharjan and Saxena, 2020; González-Arzola, 2024). This transition is accompanied by loss of NPM1 mobility, morphological changes in the nucleolus, and its functional impairment (Frottin et al., 2019).

Interestingly, stress response-driven accumulation of nucleolar lncRNAs (IGSRNA and PAPAS) was observed (Gavrilova et al., 2024; Han and Chen, 2024). Heat shock and low pH induces the formation of amyloid-like condensates in the nucleolus, known also as A-bodies. These aggregates are reversible and contain functional amyloid-like proteins associated with specific lncRNAs rich in dinucleotide repeats, transcribed from ribosomal DNA intergenic spacers (IGS) (Audas et al., 2012; Jacob et al., 2013; Wang et al., 2018; Gavrilova et al., 2024; González-Arzola, 2024; Han and Chen, 2024). However, the mechanisms ensuring stimulus-specific activation of these transcripts remain largely unknown. The stress-type specificity of nucleolar body composition also remains a mystery. Investigating the mechanisms of RNA-protein interactions in these structures, as well as the processes promoting aggregation and amyloid formation in the nucleolus, may shed light on the cellular and molecular pathways of pathological nucleolar aggregation (Gavrilova et al., 2024).

### 7.5 NPM1 is a key sensor and molecular switch in nucleolar stress

Existing evidence indicates that redox changes within the nucleolar compartment serve as a common trigger for NPM1 relocationc (Yang et al., 2018). A key event is the post-translational modification of NPM1its S-glutathionylation at Cys275 (Yang et al., 2018). This modification causes NPM1 dissociation from ribosomal DNA (rDNA) and RNA (rRNA) weakening its association with the nucleolar matrix (Yang et al., 2018). Reducing agents (e.g., DTT) or glutathione synthesis precursors (e.g., NAC), which neutralize oxidative modifications, effectively prevent stress-induced NPM1 translocation (Yang et al., 2018; Taha and Ahmadian, 2024).

Thus, the nucleolus functions as a multisensory integrator of cellular stress. The key universal mechanism of this integration is the dynamic redistribution of nucleolar proteins, particularly NPM1, which acts as a central molecular switch linking stress detection to the global rewiring of signaling pathways, including those mediated by p53, NF-κB, and protein quality control systems.

## 8 Aberrant ribosome biogenesis as a causal factor in oncogenesis

It is well known that enhanced translational capacity resulting from increased ribosome biogenesis promotes cancer development and progression (Johnson et al., 1976; Bilanges and Stokoe, 2007; White, 2008; Bursac et al., 2014). Evidence suggests that the RPL5-RPL11-MDM2-p53 pathway may regulate excessive ribosome biogenesis to prevent oncogenesis (Oskarsson and Trumpp, 2005; Van Riggelen et al., 2010; Golomb et al., 2012; Destefanis et al., 2020). Independent signaling pathways of RPL5-RPL11-MDM2-p53 and ARF-MDM2-p53 control excessive ribosome biogenesis to protect cells from MYC-induced oncogenesis (Ruggero, 2009; Macias et al., 2010; Van Riggelen et al., 2010; Destefanis et al., 2020). At the same time, RPL5-RPL11 may play a role in oncogenesis independent of the MDM2–p53 module. The ARF gene can be mutated or silenced in tumor cells (Lowe and Sherr, 2003; Montanaro et al., 2012; Maggi et al., 2014), and loss of ARF expression may be responsible for enhanced ribosome biogenesis both directly and through effects on p53 stabilization (Montanaro et al., 2012).

Increased expression of ribosomal proteins is also associated with increased proliferation and growth (Van Riggelen et al., 2010). There have been numerous instances of ribosomal protein overexpression across many different cancer types. For example, RPLP0, RPLP1, and RPLP2 levels are found to be upregulated at the RNA level in patients with gynecologic tumors, and this upregulation positively correlates with the presence of lymph node metastases in ovarian cancer (Artero-Castro et al., 2011; Weeks et al., 2019). Furthermore, the overexpression of ribosomal proteins RPS11 and RPS20, both collectively and individually, has been found to be a predictor of poor survival outcomes in patients with glioblastoma (Yong et al., 2015; Weeks et al., 2019). In colorectal cancer cell lines HCT116 and HT-29, knockdown of RPS24 was found to inhibit cell migration and proliferation (Wang et al., 2015; Weeks et al., 2019). Conversely, overexpression of RPL19 in the breast cancer cell line MCF7 rendered the cells more susceptible to stress-induced cell death (Hong et al., 2014; Weeks et al., 2019).

Although many reports identify mutations in ribosomal proteins (RPs) occurring in cancer, it is hard to prove whether these mutations are oncogenic factors. These mutations are often reported alongside mutations in tumor suppressor genes and are considered as secondary due to genomic instability. However, several studies suggest that disregulated RPs could possibly induce oncogenesis. For example, RPL5, RPL14, and RPL22 are negative regulators of c-MYC (Rao et al., 2012; Liao J. M. et al., 2014; Zhou et al., 2015). Therefore, mutations in these RPs cause c-MYC overexpression and may contribute to transformation (Rao et al., 2012; Morgado-Palacin et al., 2015). New data indicate that RPs also regulate the NF-κB family of transcription factors. Characterization of approximately 500 lines of *Danio rerio* with heterozygous mutations in recessive lethal genes for early lethality and tumor development identified 12 lines with increased cancer incidence, 11 of which carry heterozygous mutations in RP genes (Zhang and Lu, 2009). Accordingly, aberrant RP expression in mouse models can stimulate oncogenesis (Rao et al., 2012). In humans, hyperplastic and dysplastic changes in epithelial cells promote neoplastic transformation (Montanaro et al., 2012). Human tissues and organs chronically affected by inflammatory diseases display increased tumor incidence. However, a common feature of neoplastic transformation prone conditions is nucleolar hypertrophy (Derenzini et al., 2009; Montanaro et al., 2012; Jia et al., 2024). This indicates that the rate of ribosome biogenesis is increased in hyperplastic and dysplastic cells, as nucleolar size directly correlates with Pol I transcriptional activity (Derenzini et al., 1998; Montanaro et al., 2012). The upregulation of ribosome biogenesis appears to be a consequence of high metabolic demands induced by hyperproliferation. Recent data indicate that increased ribosome biogenesis rates can indeed promote cell progression toward neoplastic transformation (Montanaro et al., 2012; Penzo et al., 2019). Qualitative alterations in ribosome biogenesis can be a reason for development of human diseases, primarily a group of rare inherited disorders called ribosomopathies (Narla and Ebert, 2010). In these diseases, genes encoding factors required for ribosome production—such as ribosomal proteins or other factors involved in rRNA transcription and processing—are mutated. These disorders include congenital X-linked dyskeratosis congenita (X-DC), Shwachman-Diamond syndrome (SDS), cartilage-hair hypoplasia (CHH), Diamond-Blackfan anemia (DBA), and Treacher Collins syndrome (Narla and Ebert, 2010; Golomb et al., 2014). DBA is characterized by bone marrow failure due to inhibited differentiation of hematopoietic stem cells along the erythroid lineage (Narla and Ebert, 2010; Golomb et al., 2014). Mutations associated with DBA have been identified in eleven different genes encoding both small and large subunit RP (Boria et al., 2010; Bohnsack and Bohnsack, 2019). Ribosomopathies have diverse phenotypes, but many share common or overlapping features such as hematopoietic dysfunction (e.g., DBA, 5q syndrome, congenital dyskeratosis, Shwachman-Diamond syndrome) and/or skeletal defects (especially craniofacial) (e.g., Treacher Collins syndrome, Bowen-Conradi syndrome, and RPS23-related ribosomopathy). Understanding how defects in a fundamental and ubiquitous cellular process like ribosome synthesis cause tissue-specific human pathologies remains a major challenge, and several models have been proposed to explain these phenomena (Mills and Green, 2017; Bohnsack and Bohnsack, 2019; Emmott et al., 2019). Since mutations in genes encoding RPs or ribosome biogenesis factors disrupt ribosome assembly, they lead to p53 stabilization via the 5S RNP/MDM2 pathway described above. Indeed, many tissue-specific defects observed in ribosomopathies appear to be p53-dependent, and symptoms of ribosomopathies such as Treacher Collins syndrome and 5q syndrome can be alleviated by inhibiting p53 function (Bohnsack and Bohnsack, 2019; Emmott et al., 2019). It has been suggested that differences in p53 activation threshold or degree among cell types underlie tissue specificity in many ribosomopathies. An alternative hypothesis posits that differences in ribosome composition among cell types and/or tissues lead to specialized translation. Mutations that reduce specific RP levels or disrupt rRNA modification mechanisms may thereby reduce or alter the production of proteins particularly required in certain cell types. Another possible molecular basis for tissue-specific effects in ribosomopathies could be translational changes caused by high ribosome demand in rapidly proliferating tissues. Supporting this, DBA-associated mutations reduce ribosome levels in hematopoietic cells, so ribosome availability becomes limiting, affecting translation of a subset of mRNAs, which in turn impairs erythroid lineage commitment (Khajuria et al., 2018; Bohnsack and Bohnsack, 2019). It has been proposed that differential effects on mRNA translation caused by ribosome scarcity arise from specific mRNA features (e.g., highly structured 5′-UTRs), rendering these transcripts especially sensitive to reduced ribosome concentrations. It is likely that differential p53 responses to ribosome assembly defects and changes in translation profiles caused by limited ribosome production and/or assembly of specialized ribosomes contribute to ribosomopathy phenotypes to varying degrees (Bohnsack and Bohnsack, 2019). DBA patients are predisposed to develop myelodysplastic syndrome (MDS), acute myeloid leukemia, and solid tumors (Vlachos et al., 2012; Golomb et al., 2014). The most common genetic alterations in DBA are found in the RPS19 gene, but mutations and deletions have also been identified in several other RP genes (Narla et al., 2011; Golomb et al., 2014). Haploinsufficiency of RPS14 and RPSA has been detected in 5q syndrome, acquired somatic deletion of chromosome 5q, and isolated congenital asplenia (Vlachos et al., 2012; Golomb et al., 2014), respectively. Recent studies on animal models of ribosomopathies and patients confirm the role of p53 in mediating specific pathological manifestations of these disorders (Bursac et al., 2014; Golomb et al., 2014). Interestingly, X-DC, SDS, CHH, and DBA are characterized by high cancer incidence, supporting the hypothesis that specific qualitative defects in ribosome biogenesis can also induce oncogenesis (Narla and Ebert, 2010). It has been proposed that changes in the complex ribosome structure may be responsible for alterations in mRNA translation relevant to neoplastic transformation. For example, X-DC is caused by mutations in the gene encoding the ubiquitin ligase dyskerin (DKC1) and is characterized by progressive proliferative tissue failure associated with an 11-fold increased risk of malignancy compared to the general population (Narla and Ebert, 2010; Poortinga et al., 2011; Taoka et al., 2018). The DKC1 product, dyskerin, is a key protein involved in small nucleolar ribonucleoproteins (snoRNPs) involved in rRNA processing (Meier, 2006; Kiss et al., 2010; Böğürcü-Seidel et al., 2023). It is also necessary for site-specific conversion of uridine to pseudouridine in rRNA molecules (Meier, 2006; Kiss et al., 2010; Böğürcü-Seidel et al., 2023). Pseudouridines are located within specific ribosome domains important for tRNA and mRNA binding, and loss of pseudouridine incorporation into rRNA may impair translation of specific mRNAs (Strunk et al., 2012; Ghalei et al., 2017; Sharma et al., 2017). Altered rRNA modifications can trigger tumorigenesis or promote oncogenesis. Aberrant expression of small nucleolar RNAs (snoRNAs) affects the methylation process (Elhamamsy et al., 2025; Lafontaine, 2015; Gay et al., 2022). For instance, increased expression of snoRNA SNORD42A, responsible for 2′-O-methylation of U116 in 18S rRNA, is observed in acute myeloid leukemia (AML) patients (Elhamamsy et al., 2025; Marcel et al., 2013). Breast cancer samples show variability in 2′-O-methylation of rRNA, which correlates differentially with tumor subtype and malignancy grade (Elhamamsy et al., 2025; Marcel et al., 2020). When tumor cells experience stress, such as hypoxia, rRNAs acquire different methylation patterns and generate specialized ribosome subpopulations capable of internal ribosome entry site (IRES)-mediated translation (Elhamamsy et al., 2025; Metge et al., 2020). In normal cells, the expression of the key rRNA methyltransferase fibrillarin is regulated by p53. Mutations or alterations in p53 affect fibrillarin levels and activity, resulting in hypermodified rRNA, with 2′-O-methylation occurring at sites normally unmethylated in p53-positive cells (Sharma et al., 2017; Marcel et al., 2018), potentially causing decreased translation accuracy and enhanced IRES-mediated translation of several oncogenes (Elhamamsy et al., 2025; Macias et al., 2010; Marcel et al., 2013; 2018). Thus, the paradoxical observation that RP misregulation characterized by hypoproliferation leads to hyperproliferative disease such as cancer has several explanations: selective pressure bypassing p53, direct regulation of oncogenes and tumor suppressors by RPs, and preferential mRNA translation including the existence of specialized “oncoribosomes.” Changes in ribosome concentration may also lead to selective mRNA translation and oncogenesis. Besides p53 homeostasis regulation, aberrant RP expression may cause p53 mutations.

Some ribosome biogenesis factors, such as nucleolin (NCL) (Table 1), also play a role in cancer development and metastasis. NCL is not exclusively localized to the nucleolus; it can also be found in the cytoplasm and on the cell outer membrane. Various ligands are capable of binding to cell surface NCL and influencing the physiological functions of the cell (Chen and Xu, 2016). The interaction of endostatin with NCL on the surface of endothelial cells potentiates an anti-angiogenic response and inhibits tumor growth (Weeks et al., 2019).

## 9 Ribosome biogenesis, cell differentiation, and developmental pathologies

Most nervous system pathologies are caused by abnormal protein synthesis, and ribosome dysfunction disrupts the homeostasis of neurons and glial cells (Drokhlyansky et al., 2020; Scheurer et al., 2021; Jiao et al., 2023). Accumulating data indicate that disrupted ribosome biogenesis is involved in aging and neurodegeneration (Jiao et al., 2023). Moreover, dysregulation of ribosome biogenesis is neuropathogenic in the context of the developing nervous system (Hetman and Slomnicki, 2019). Depending on the developmental period/cell type, the pathological consequences can range from microcephaly caused by loss of functions such as ID or ASD to neurodegeneration (Hetman and Slomnicki, 2019). In recent years, there has been an increase in the number of diseases identified as novel congenital ribosomopathies associated with severe developmental pathologies. These extremely rare disorders are characterized by mutations in ribosomal proteins or in factors involved in ribosome biogenesis (Venturi and Montanaro, 2020). These ribosomopathies are heterogeneous diseases, manifesting as generalized multisystemic symptoms or more specific manifestations selective for a single tissue or organ. A striking example of a multisystemic ribosomopathy is the rare and severe Bowen-Conradi syndrome (BCS), which is characterized by intellectual disability, microcephaly, micrognathia, a prominent nose, rocker-bottom feet, and flexion contractures of the joints (Lowry et al., 2003; Armistead et al., 2014; 2015; Venturi and Montanaro, 2020), and which leads to death in early childhood. Its cause is a specific mutation in the gene for the Essential for Mitotic Growth 1 (EMG1) protein. EMG1 is a methyltransferase necessary for the proper maturation of 18S rRNA (Armistead et al., 2014). The mutation leads to protein destabilization and a substantial decrease in its levels in patient-derived fibroblasts and lymphoblasts. Furthermore, the mutation causes a delay in the processing of the 18S rRNA precursor and disrupts the normal intracellular localization of the mutant EMG1 protein: its recruitment to the nucleolus is reduced, while it accumulates in abnormal nuclear foci and is subsequently degraded via the proteasome (Warda et al., 2016; Menjivar-Vallecillo et al., 2025). This results in significantly reduced proliferation and a G2/M phase arrest, which impedes normal cell entry into mitosis. Interestingly, however, steady-state levels of mature 18S rRNA and overall protein synthesis rates may remain unchanged (Armistead et al., 2014; 2015).

Tissues with rapid proliferation, such as the neuroepithelium of the developing embryo where EMG1 expression is particularly high, demonstrate a special vulnerability to EMG1 dysfunction. The reduced proliferation of neuroepithelial cells and the underlying mesenchyme likely explains the severe nervous system malformations in patients and the impaired neural tube closure—the precursor to the central nervous system—observed in mouse models (Armistead et al., 2015). This developmental defect may be directly linked to the loss of EMG1’s methyltransferase activity, given the established importance of methylation processes (particularly the folate cycle) for neurulation. As a potential therapeutic approach for BCS, the use of S-adenosylmethionine (SAM)—a universal methyl group donor and a cofactor for EMG1—is being considered (Armistead et al., 2015).

## 10 Conclusion

The nucleolus is not just a “factory” for assembling ribosomes, but a dynamic and multifunctional cellular regulatory hub, influencing key processes from cell cycle control and proliferation to decisions regarding differentiation or cell death. Consequently, dysfunction in nucleolar structure and activity is frequently central to severe pathologies, particularly oncological and neurodegenerative disorders, highlighting its critical role in maintaining normal cellular physiology.

At the core of this intricate regulatory network are two key opposing factors: the MYC oncogene and the p53 tumor suppressor. MYC acts as a master driver of cellular growth, potently stimulating the production of all ribosomal components, thereby directing the cell toward division. MYC coordinately enhances the activity of all three RNA polymerases. However, critical questions regarding its function remain open. For instance, how is the balance precisely regulated between MYC’s pro-oncogenic activity and its ability to activate suppressor pathways through excessive ribosome biogenesis stimulation? What are the subtle mechanisms determining whether MYC overexpression leads to uncontrolled proliferation or, conversely, to the activation of p53-dependent apoptosis?

In contrast to MYC, p53 is activated in response to various stresses, including DNA damage or disruptions in the ribosome assembly process itself. p53 activation leads to cell cycle arrest or apoptosis, preventing the division of damaged cells. However, the mechanism of its activation specifically during nucleolar stress remains a subject of debate and is represented by two primary, non-mutually exclusive models.

The first model, relocalization, posits that stress causes nucleolar structural reorganization, resulting in the translocation of key proteins, such as ribosomal proteins RPL5 and RPL11, into the nucleoplasm. There, they bind to MDM2 and directly inhibit its ability to ubiquitinate p53 for degradation, leading to the accumulation of active p53. The second model, direct nucleolar control, focuses on the nucleolus normally serving as a specialized compartment for efficient p53 ubiquitination. According to this model, stress does not necessarily cause massive protein export but rather disrupts the very organization of p53 ubiquitination within the nucleolus allowing p53 exit in a stable form.

It is challenging to definitively prove that the observed relocalization of RPL11/RPL5 is the cause rather than a consequence of p53 stabilization or general nucleolar restructuring. Not all studies observe massive ribosomal protein release before p53 stabilization. The release of ribosomal proteins in the first model could simultaneously trigger nucleolar homeostasis disruptions described by the second model. Both processes may operate in parallel, amplifying the response. The shuttling of p53/MDM2 through the nucleolus and the ubiquitination process are extremely rapid and dynamic, making them technically difficult to capture and measure without perturbing the entire system.

Both models primarily focus on MDM2. However, other E3 ubiquitin ligases for p53 exist, alongside alternative degradation pathways (e.g., calpain-dependent). Their potential contribution under stress conditions, which may vary, is seldom accounted for. Furthermore, the oligomeric state of p53 critically influences its degradation, localization, and activity. Neither model fully explains how nucleolar stress affects this status and how it integrates into the activation mechanism. The hypothesis regarding oligomer transition within the nucleolus remains unconfirmed. Determining which model predominates under different stress conditions or how they cooperatively amplify the signal is a significant unresolved challenge.

The search for and study of p53-independent mechanisms controlling the cell cycle in response to ribosome assembly defects is of particular importance. Several alternative pathways have been identified, such as those involving stabilization of cyclin-dependent kinase inhibitors or suppression of transcription factors. However, it remains incompletely resolved which mechanism predominates under various stress conditions. Understanding these subtle mechanisms could form the basis for developing therapies against tumors where the canonical p53 pathway is already dysfunctional.

A crucial aspect of modern understanding is the recognition of ribosome heterogeneity, stemming from variations in rDNA sequences, tissue-specific post-transcriptional rRNA modifications, differences in ribosomal protein composition, and their post-translational modifications. A key unresolved question is whether cells purposefully produce distinct ribosome populations for optimal translation of specific mRNA sets depending on tissue type, developmental stage, or external conditions—supported by evidence of tissue-specific ribosome variants, clear ribosomopathy phenotypes, and links between specific modifications and the translation of particular transcripts. Alternatively, ribosome heterogeneity may be a byproduct of inaccuracies in the biogenesis process, lacking significant functional relevance, with observed effects (as in ribosomopathies) explained by a general reduction in ribosome quantity/quality, activation of stress pathways, and global translational impairment. The signal for specialization might be statistically weak against the background of the overall ribosome pool.

To date, primarily correlations between specific variant expression and tissue types have been established. It is not proven that these variants are causally necessary and sufficient for shaping a tissue-specific proteome. It is extremely difficult to isolate and study the functional properties of pure ribosome populations containing only one specific rRNA variant in a living cell. Separating the effect of an rRNA variant from confounding factors—such as specific ribosomal protein sets, modifications, and translational factor activities—is challenging. How is the transcription of specific rDNA variants regulated in a given cell? What epigenetic marks or specific factors govern this? Furthermore, it is unclear what contributes more to ribosome “specialization”—rRNA sequence, its modifications, or ribosomal protein composition? How are these regulatory layers coordinated?

Regardless of the precise mechanism of p53 activation and the functional significance of ribosome heterogeneity, the ultimate outcome of system imbalance is malignant transformation. This often arises from MYC hyperactivation or p53 function loss, making the ribosome biogenesis process itself a vulnerable and promising therapeutic target. The investigation of p53-independent cell cycle control mechanisms activated in response to disrupted ribosome assembly is thus of paramount importance.

In conclusion, ongoing research continues to unravel the molecular complexity of the nucleolus. Understanding the dynamic balance between MYC and p53, the mechanisms of the stress response, and the implications of ribosome heterogeneity not only deepens our fundamental knowledge of cell biology but also paves the way for developing novel, more targeted therapeutic strategies against diseases rooted in nucleolar dysfunction, particularly cancer.
